# Reconstructing Genome-Wide Protein–Protein Interaction Networks Using Multiple Strategies with Homologous Mapping

**DOI:** 10.1371/journal.pone.0116347

**Published:** 2015-01-20

**Authors:** Yu-Shu Lo, Sing-Han Huang, Yong-Chun Luo, Chun-Yu Lin, Jinn-Moon Yang

**Affiliations:** 1 Institute of Bioinformatics and Systems Biology, National Chiao Tung University, Hsinchu, Taiwan; 2 Department of Biological Science and Technology, National Chiao Tung University, Hsinchu, Taiwan; 3 Center for Bioinformatics Research, National Chiao Tung University, Hsinchu, Taiwan; Texas A&M University, UNITED STATES

## Abstract

**Background:**

One of the crucial steps toward understanding the biological functions of a cellular system is to investigate protein–protein interaction (PPI) networks. As an increasing number of reliable PPIs become available, there is a growing need for discovering PPIs to reconstruct PPI networks of interesting organisms. Some interolog-based methods and homologous PPI families have been proposed for predicting PPIs from the known PPIs of source organisms.

**Results:**

Here, we propose a multiple-strategy scoring method to identify reliable PPIs for reconstructing the mouse PPI network from two well-known organisms: human and fly. We firstly identified the PPI candidates of target organisms based on homologous PPIs, sharing significant sequence similarities (joint *E*-value ≤ 1 × 10^−40^), from source organisms using generalized interolog mapping. These PPI candidates were evaluated by our multiple-strategy scoring method, combining sequence similarities, normalized ranks, and conservation scores across multiple organisms. According to 106,825 PPI candidates in yeast derived from human and fly, our scoring method can achieve high prediction accuracy and outperform generalized interolog mapping. Experiment results show that our multiple-strategy score can avoid the influence of the protein family size and length to significantly improve PPI prediction accuracy and reflect the biological functions. In addition, the top-ranked and conserved PPIs are often orthologous/essential interactions and share the functional similarity. Based on these reliable predicted PPIs, we reconstructed a comprehensive mouse PPI network, which is a scale-free network and can reflect the biological functions and high connectivity of 292 KEGG modules, including 216 pathways and 76 structural complexes.

**Conclusions:**

Experimental results show that our scoring method can improve the predicting accuracy based on the normalized rank and evolutionary conservation from multiple organisms. Our predicted PPIs share similar biological processes and cellular components, and the reconstructed genome-wide PPI network can reflect network topology and modularity. We believe that our method is useful for inferring reliable PPIs and reconstructing a comprehensive PPI network of an interesting organism.

## Introduction

To investigate protein–protein interaction (PPI) networks is one of the crucial steps toward understanding the biological functions of a cell [1–3]. The PPIs generated by high throughput experimental methods (e.g., yeast two-hybrid screening [4,5] and co-affinity purification [6]) have rapidly increased in number and they have been collected in PPI databases (e.g. BioGRID [7] and IntAct [8]). However, these experimental PPIs were distributed on several well-studied organisms, such as *H. sapiens* (67,596 PPIs) and *S. cerevisiae* (235,367 PPIs), and the number of experimental PPIs was 7,736 for *Mus musculus* according to five public databases (e.g. BioGRID and IntAct) [7–11]. As these PPIs continuously grow in size, they have become increasingly useful for reconstructing PPI networks in an interesting organism.

For an interesting organism, several computational methods, such as PathBlast [12,13] and interologs [14,15], have been proposed to identify PPIs through the homologs and orthologs. However, most of these methods focus on predicting PPIs in a target organism mapped from one source organism. Recently, we have provided the PPI family, which consists of homologous PPIs, and the protein complex family for interesting organisms by mapping from multiple organisms [16–19]. These predicted PPIs and families using sequence-based methods provided the opportunity for reconstructing the PPI network of a target organism. However, these sequence-based methods (e.g., generalized interolog mapping and interolog mapping) have two major disadvantages: high false positive-rates for generalized interolog mapping and low coverage rates for interologs method [15]. Our previous works used structure complexes to improve the prediction accuracy and the inferred homologous PPIs by considering the binding models and atomic interactions [17,20]. However, the number of X-ray structure complexes is much smaller than the number of experimental PPIs recorded in databases.

To address these issues, we propose a multiple-strategy scoring method, combining sequence similarities (*S_sim_*), normalized ranks (*S_rank_*), and conservation scores (*S_con_*), to identify reliable PPIs for reconstructing biological networks using homologous PPIs across multiple organisms. Our multiple-strategy score is able to avoid the disadvantages (e.g. high false positive rate and low coverage rate) of sequence-based mapping methods to predict reliable genome-wide PPIs for an interesting organism. Our experimental results show that 72.0% of PPIs with high *S_rank_* values (*S_rank_* > 0.9) are orthologous protein pairs and share high functional similarities. In addition, we found that the family sizes of two interacting proteins are highly correlated with the number of predicted PPI candidates using generalized interolog mapping because these proteins in a family often are homologous and share high sequence similarity. *S_rank_* is able to avoid the influence of the protein family size and protein length to significantly improve PPI prediction accuracy and to reflect the biological functions. Furthermore, the top-ranked and conserved PPIs (*S_con_*) are often orthologous/essential interactions and share functional similarity. Finally, we can use these reliable predicted PPI to reconstruct a comprehensive PPI network of a target organism. This network is a scale-free network and can reflect the biological functions and module properties. We believe that our method is useful for reconstructing genome-wide PPI networks in an interesting organism.

## Materials and Methods

### Overview

For given known PPIs in the source organisms (i.e., *H. sapiens* and *D. melanogaster*), we derived their homologous PPIs to construct genome-wide PPI networks in a target organism (e.g., *S. cerevisiae* or *M. musculus*). Our previous studies have proposed the method for inferring the homologous PPIs (e.g., A′-B′) in the target organism for the known PPI (A-B) in the source organism [16,17]. The concept of homologous PPIs is briefly described as follows: (1) Proteins A′ and B′ are the homologs of A and B, respectively; (2) The PPIs A′-B′ and A-B share significant interface similarity.

**Figure 1:**
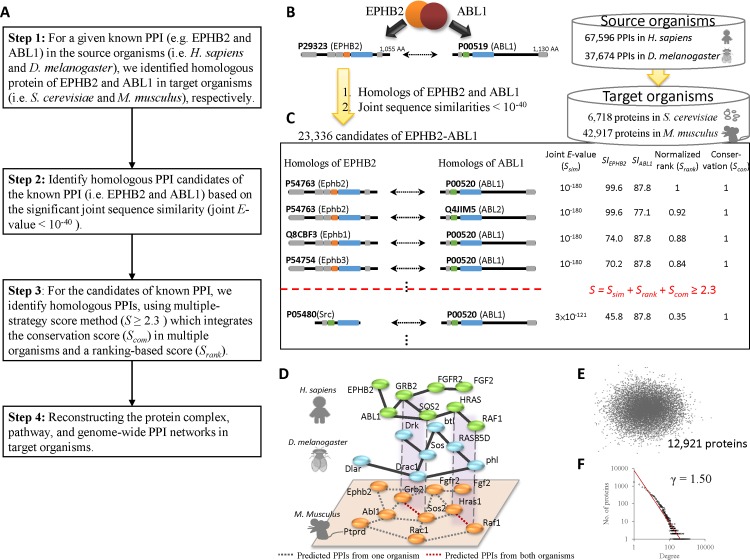
Overview of reconstructing genome-wide PPI networks using the multiple-strategy scoring method and homologous mapping. (A) Main procedure. (B) For a known PPI (e.g. EPHB2-ABL1) in the source organisms, the PPI candidates (joint *E*-value ≤ 1 × 10^−40^) in the target organism are identified by searching the whole genome protein sequences using BLASTP. (C) These PPI candidates are scored by using the multiple-strategy scoring system (*S*), including the joint sequence similarity (*S_sim_*), normalized rank (*S_rank_*), and consensus (*S_con_*). The candidates meeting the interaction score criterion (*S* ≥ 2.3) are considered as the predicted PPIs. (D) Part of the network is reconstructed by some predicted PPIs in the target organism (*M. musculus*) from known PPIs in two source organism (*H. sapiens* and D. *melanogaster*). The grey dashed lines denote the PPIs predicted from a single source organism. The red dashed lines denote the intersection PPIs predicted from both organisms. (E) The reconstructed mouse PPI network based on these predicted PPIs. (F) The distribution of degrees of this reconstructed mouse PPI network, which is a weak scale-free network (γ = 1.5).

To infer the comprehensive PPI network of the target organism, we propose a multiple-strategy score method which integrates a ranking-based score and a conservation score in multiple organisms. Fig. 1A shows the main procedure of reconstructing genome-wide PPI networks in target organisms. For a given known PPI (A-B, e.g. EPHB2-ABL1), first, we identify its homologous PPI candidates (e.g., A′-B′) by considering the homologous proteins (*E*-value ≤ 1 × 10^−10^) of the pair proteins and the joint sequence similarities (joint *E*-value ≤ 1 × 10^−40^) in target organisms (e.g., *M. musculus*) by using BLASTP (Fig. 1B). The joint *E*-value (JE) is defined as the geometric mean of individual *E*-values of a protein pair [16,21]. Second, we used the multiple-strategy scoring method to calculate the interacting score (*S*, Fig. 1C) of the PPI candidate (A′ and B′), which is defined as

$$S=w_{1}S_{sim}+w_{2}S_{rank}+w_{3}S_{con},$$ (Eq. 1)

where *S_sim_* is the normalized joint sequence similarity, *S_rank_* is the normalized rank, and the *S_con_* is the conserved score based on the multiple organisms. For these three terms (*S_sim_*, *S_rank_*, and *S_con_*), the score ranges from 0 to 1, and the total score *S* ranges from 0 to 3. The *w_1_*, *w_2_*, and *w_3_* values were yielded by testing various values ranging from 0 to 1 on the YD set (S1 Fig. and S1 Table). Here, we set *w_1_, w_2_*, and *w_3_* to 1. The *S_sim_* is given as

$$S_{sim}=\frac{\sqrt{\left(-\log_{10}E_{A'})\times (-\log_{10}E_{B'}\right)}}{\sqrt{\left(-\log_{10}E_{A})\times (-\log_{10}E_{B}\right)}},$$ (Eq. 2)

where *E_A′_* is the BLAST *E*-value between A and A′, *E_B′_* is the BLAST *E*-value between B and B′, and *E_A_* and *E_B_* are the BLAST *E*-values when aligning A to A and B to B, respectively. Because the maximum BLAST *E*-value depends on the protein length, we used *E_A_* and *E_B_* as the maximum values to normalize joint sequence similarity (0 ≤ *S_sim_* ≤ 1). *S_rank_* is calculated as

$$S_{rank}=1-\frac{\log(r_{A'-B'})}{\log(r_{\max})},$$ (Eq. 3)

where *r_A′-B′_* is the rank of candidate A′-B′ based on the joint sequence similarity (i.e. *S_sim_*); *r_max_* is the total number of PPI candidates derived from known PPI A-B. For a given PPI A-B, if the homologous protein pairs A_1_-B_1_ and A_2_-B_2_ have the same value of *S_sim_*, we used the joint sequence identity ($joint-SI=\sqrt{SI_{1}\times SI_{2}}$) to rank the pairs. The sequence identity (SI) is determined using BLASTP. Finally, *S_con_* is defined as

$$S_{con}=E_{A'-B'}^{H-\mathrm{Target}}+E_{A'-B'}^{D-\mathrm{Target}},$$ (Eq. 4)

where $E_{A'-B'}^{H-\mathrm{Target}}$ and $E_{A'-B'}^{D-\mathrm{Target}}$ are set to the normalized evolutionary distances, if the candidate A′-B′ can be derived from the source organisms *H. sapiens* and *D. melanogaster*, respectively. $E_{A'-B'}^{H-\mathrm{Target}}$ or $E_{A'-B'}^{D-\mathrm{Target}}$ is set to 0, if A′-B′ is unable to be predicted from the respective source organism. This distance was obtained based on the phylogenetic tree with 273 species proposed by InParanoid [22]. According to this phylogenetic tree, the normalized evolutionary distances from *H. sapiens* and *D. melanogaster* to *M. musculus* are 0.765 and 0.235, respectively. In addition, the distance between *H. sapiens* and *S. cerevisiae* is equal to the distance between *D. melanogaster* and S. cerevisiae. For example, the *S_con_* value of candidate Sos1-Grb2 in *M. musculus* is 1 because this PPI was derived from both known PPIs, SOS2-GRB2 in *H. sapiens* and Sos-Drk in *D. melanogaster*. On the other hand, the *S_con_* value of candidate Fgfr2-Fgf1 in *M. musculus* is 0.765, because it was derived from FGFR2-FGF1 in *H. sapiens* only (Fig. 1D). Finally, the PPI candidates with significantly high interaction scores (*S* ≥ 2.3) were selected to reconstruct the PPI network in the target organism (e.g. *M. musculus*, Fig. 1C, 1D, and 1E). We then analyzed this PPI network using network topology (Fig. 1F), biological functions and processes. The reconstructed PPI network in *S. cerevisiae* contains 476 proteins and 1,094 PPIs; 12,921 proteins and 153,852 PPIs in the reconstructed mouse PPI network. The degree exponent γ is 1.5 in the reconstructed mouse PPI network which is consistent with the architecture (i.e., weak scale-free network properties) of some cellular networks [23,24].

### Source and target organisms

We collected 37,674 and 67,596 PPIs, recorded in five public databases [7–11], of *D. melanogaster* and *H. sapiens*, respectively. These two source organisms have been well studied. We then selected *S. cerevisiae* and *M. musculus*, which are the common experimental organisms, as the target organisms. The experimental PPIs of *S. cerevisiae*, collected from five public databases, were used for evaluating the accuracy and characteristics of our multiple-strategy scoring method. The genome-wide protein sequence data of the source and target organisms were collected from the UniProt database [25].

### Gold standard positives and negative cases

To evaluate the reliability of homologous PPIs derived from our method and scoring function, we collected two datasets, termed YD (*S. cerevisiae*) and MD (*M. musculus*), as the gold standard positive and negative sets. The positive PPIs in YD and MD sets are experimentally derived PPIs. The YD set consists of 928 positive PPIs, recorded as the core subset in the DIP database [10], and 23,014 negative PPIs, as defined by Jansen et al. [26]. For the MD set, 3,354 positive PPIs were collected from the five public databases [7–11]. The negative PPIs were defined by using the relative specificity similarities (*RSS_BP_* and *RSS_CC_*) of biological process (BP) and cellular component (CC), as proposed by Wu et al [27] (S2 Fig). Here, the 42,665 PPIs, for which *RSS_BP_* < 0.4 and *RSS_CC_* < 0.4, were considered as the negative cases.

## Results and Discussions

### Homologous mapping across multiple species

We utilized a multiple-strategy scoring method to improve the accuracies of generalized interolog mapping and interolog. First, we evaluated the prediction accuracy of these three methods on a well-studied organism, *S. cerevisiae*, based on the YD set. Based on the joint sequence similarities (joint E-value ≤ 1 × 10^−40^), the generalized interolog mapping identified 90,597and 35,388 PPI candidates from the source organisms, *H. sapiens* and *D. melanogaster*, respectively. Among these total 106,825 non-redundant PPI candidates from 532,218 predicted PPIs, 928 and 23,014 candidates were recorded in the gold standard positive and negative set, respectively, based on the YD set. Conversely, the interolog method (considering the first rank PPI only) inferred 9,543 candidates, including 510 positive cases and 1,723 negative cases.

**Figure 2:**
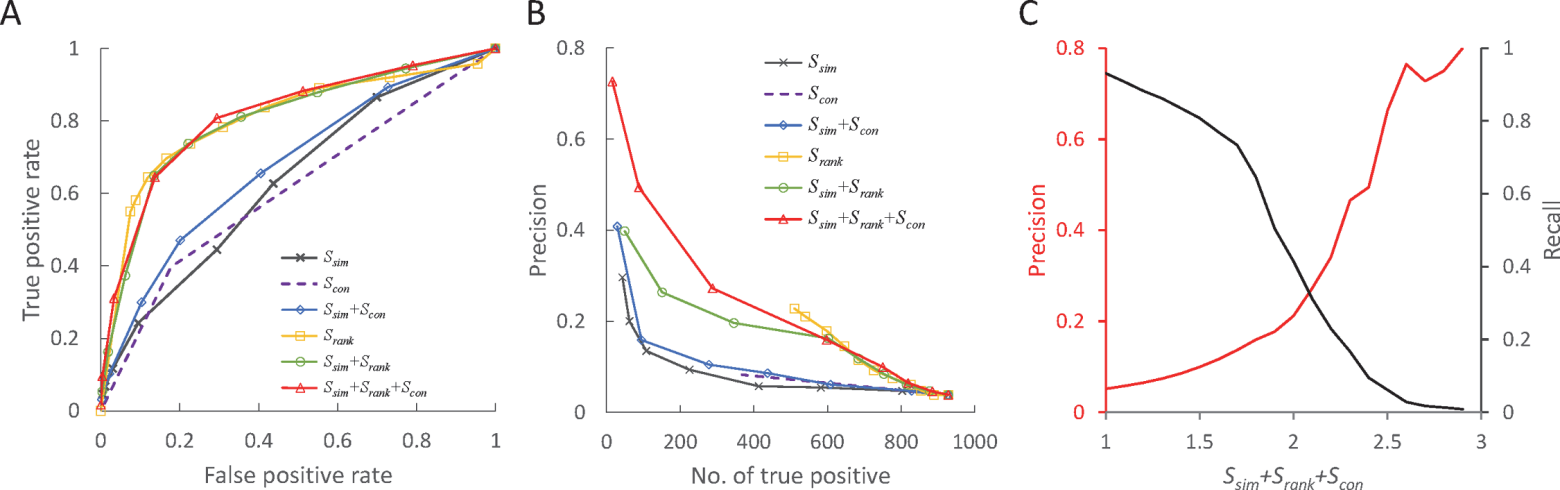
Performance of the multiple-strategy scoring method on the YD set. (A) The ROC curves of six scoring combinations, including joint sequence similarity (*S_sim_*), normalized rank (*S_rank_*), conserved score (*S_con_*), *S_sim_* + *S_rank_*, *S_sim_* + *S_con_*, and *S_sim_* + *S_rank_* + *S_con_*, on the YD set for the target organism, *S. cerevisiae*. (B) The relationship between the number of true-positive cases and the precision of these six scoring methods. Among these six combinations, *S_sim_* + *S_rank_* + *S_con_* is the best. (C) The relationship between recall (black line) and precision (red line) of the multiple-strategy scoring method (*S_sim_* + *S_rank_* + *S_con_*) by mapping two organisms (i.e. *D. melanogaster* and *H. sapiens*) to *S. cerevisiae*.

We utilized the multiple-strategy scoring method, *S* = *S_sim_* + *S_rank_* + *S_com_*, to evaluate these 106,825 PPI candidates. Fig. 2A illustrates the ROC curves (i.e. true positive and false positive rates) of six combination scoring methods (i.e. *S_sim_*, *S_rank_*, *S_sim_* + *S_rank_*, *S_sim_* + *S_con_*, and *S_sim_* + *S_rank_* + *S_con_*) for the YD set. The generalized interolog mapping considered only the joint sequence similarity (*S_sim_*). We found that combining the normalized rank (*S_rank_*) or conserved score (*S_con_*) with generalized interolog mapping (*S_sim_*) is able to significantly improve the performance for the YD set (blue and green lines in Fig. 2A).

Furthermore, we used the precision and number of true positive cases to evaluate the performance of these six scoring methods (Fig. 2B). Here, the precision is defined as *TP* / (*TP*+*FP*), where *TP* and *FP* are the numbers of true-positive and false-positive cases, respectively. The normalized rank (*S_rank_*, yellow line) is significantly better than joint sequence similarity (*S_sim_*) and a combination of these two scores (*S_sim_* + *S_rank_*) outperforms the combination of combining *S_sim_*+*S_con_*. We found that our scoring function (*S_sim_* + *S_rank_* + *S_con_*, red line in Fig. 2B) achieves the highest precision when these six scoring methods predicted the same number of true positive cases. Considering either normalized rank or conserved score with normalized joint sequence similarity improve the predictive precision compared with using only normalized joint sequence similarity. In addition, we evaluated the performance of six scoring combinations on the MD set, which consists of 3,354 and 42,665 candidates in the gold standard positive and negative set, respectively. Among these combinations, the multiple-strategy score (*S_sim_* + *S_rank_* + *S_con_*) achieves the best result on MD set (S3 Fig.).

Based on these 106,825 candidates (928 positive and 23,014 negative cases) in the YD set, we used the recall and precision to determine the threshold of our scoring function to infer the PPIs in the target organism from known PPIs in multiple source organisms (Fig. 2C) using homologous mapping. Here, we use F_0.5_-measure ($F_{0.5}=\frac{(1+0.5^{2})\times \mathrm{precision}\times \mathrm{recall}}{0.5^{2}\times \mathrm{precision}+\mathrm{recall}}$), which puts more emphasis on precision than recall, to assess our scoring function. The highest F_0.5_-measure value is 0.34 when *S* is 2.3. Here, we set the threshold of *S* to 2.3. These experimental results show that our scoring method can achieve high accuracy for predicting PPIs in a target organism from multiple organisms. These PPIs provide the opportunity for reconstructing a comprehensive PPI network for exploring the cell behavior of a target organism.

### Normalized joint sequence similarity score *S_sim_*

We further investigated the contribution of normalized joint sequence similarity in our scoring function. The generalized interologs used the certain threshold (e.g. *E*-value < 1 × 10^−70^) of joint sequence similarity to identify the PPI candidates [15]. Based on this threshold, the generalized interologs often yielded high false positive rate and low accuracy because the proteins have various sequence lengths and domain architectures. For example, the human EPHB2 (UniProt accession number: P29323) has 1,055 amino acids, including several Pfam domains, such as one Pkinase_Tyr domain, one EphA2_TM domains, and the other areas (Fig. 1C) [28]. Among the 169 homologs (*E*-value < 1 × 10^−10^) of EPHB2 in *M. musculus*, 14 homologous proteins, such as Ephrin receptor A and B families, have the highest sequence similarity (i.e., *E*-value = 0). For another example, the human protein ABL1 (tyrosine-protein kinase ABL1, UniProt accession number: P00519) consists of 1,130 amino acids and four Pfam domains (Fig. 1C). Based on ABL1 and the certain threshold, there are 257 homologous proteins in *M. musculus*. Based on the threshold (*E*-value < 1 × 10^−70^) of the joint sequence similarity, general interologs method yielded 4,604 homologous PPIs of EPHB2-ABL1 and the highest joint sequence similarity is 1 × 10^−180^ in *M. musculus*. For the PPI EPHB2-ABL1, the interacting domains are EphA2_TM (orange) and SH2 (green) (Fig. 1C).

On the other hand, the number of homologous proteins of short proteins is often significantly reduced based on the same threshold. For example, the human protein, small nuclear ribonucleoprotein E (SNRPE; UniProt accession number: P62304), has 92 amino acids and a single homologous protein (Snrpe; UniProt accession number: P62305 and *E*-value is 4 × 10^−49^) in *M. musculus* using BLASTP even though the sequence identity is 100% between these two proteins. Therefore, we cannot infer homologous PPIs in *M. musculus* for several known human PPIs (e.g. SNRPG-SNRPE and SNRPE-SNRPF) under the certain higher threshold (i.e., *E*-value < 1 × 10^−70^).

To address this issue, we used the normalized joint sequence similarity *S_sim_* (Eq. 2). For example, according to the BLASTP *E*-value between SNRPE and SNRPE is 3 × 10^−68^, the normalized sequence similarity between the SNRPE and Snrpe should be the log_10_ (4 × 10^−49^) / log_10_ (3 × 10^−68^). Based on this *S_sim_* value, two known human PPIs, SNRPG-SNRPE and SNRPE-SNRPF, could infer the two homologous PPIs (i.e. Snrpg-Snrpe and Snrpe-Snrpf) with *S_sim_* > 0.87 and S > 2.64 (S2 Table). SNRPG and SNRPE are key components of the Sm core complex which plays an essential role in the formation of small nuclear ribonucleoproteins (snRNPs) by binding to small nuclear RNAs [29]. The Sm core complex is a conserved complex in mammalians. *S_sim_* can recapture the potential homologous PPIs and reduce the disadvantages of the joint sequence similarity, which depends on the protein sequence length.

### Normalized rank score *S_rank_*

Based on the certain threshold of the joint sequence similarity, generalized interologs mapping often infers greater numbers of homologous PPIs from known PPIs with large families. For example, the general interologs method inferred 6,838 homologous PPIs in mouse from the PPI ABL1-EPHB2 in human because these proteins have a kinase domain and a large protein family (i.e., many protein members in this family; Fig. 1C). Based on the PPI candidates derived from the known PPIs in the YD and MD sets, we observed several results: 1) the number of homologous PPI candidates in *M. musculus* is significantly greater than that in *S. cerevisiae* (S4 Fig.); 2) the prediction accuracy of generalized interologs often decreases as the number of PPI candidates increases; 3) a known PPI often predicts more PPI candidates in mouse than in yeast when paralogs are relatively abundant on *M. musculus*. For example, ABL1 and EPHB2 are tyrosine-protein kinases (in human), and we can find 257 and 169 homologous proteins, respectively, with kinase domain in *M. musculus* using BLATP. We suggest that a great number of paralogs would result in a rapidly increasing number of PPI candidates and subsequently poor prediction accuracy by using joint sequence similarity.

**Figure 3:**
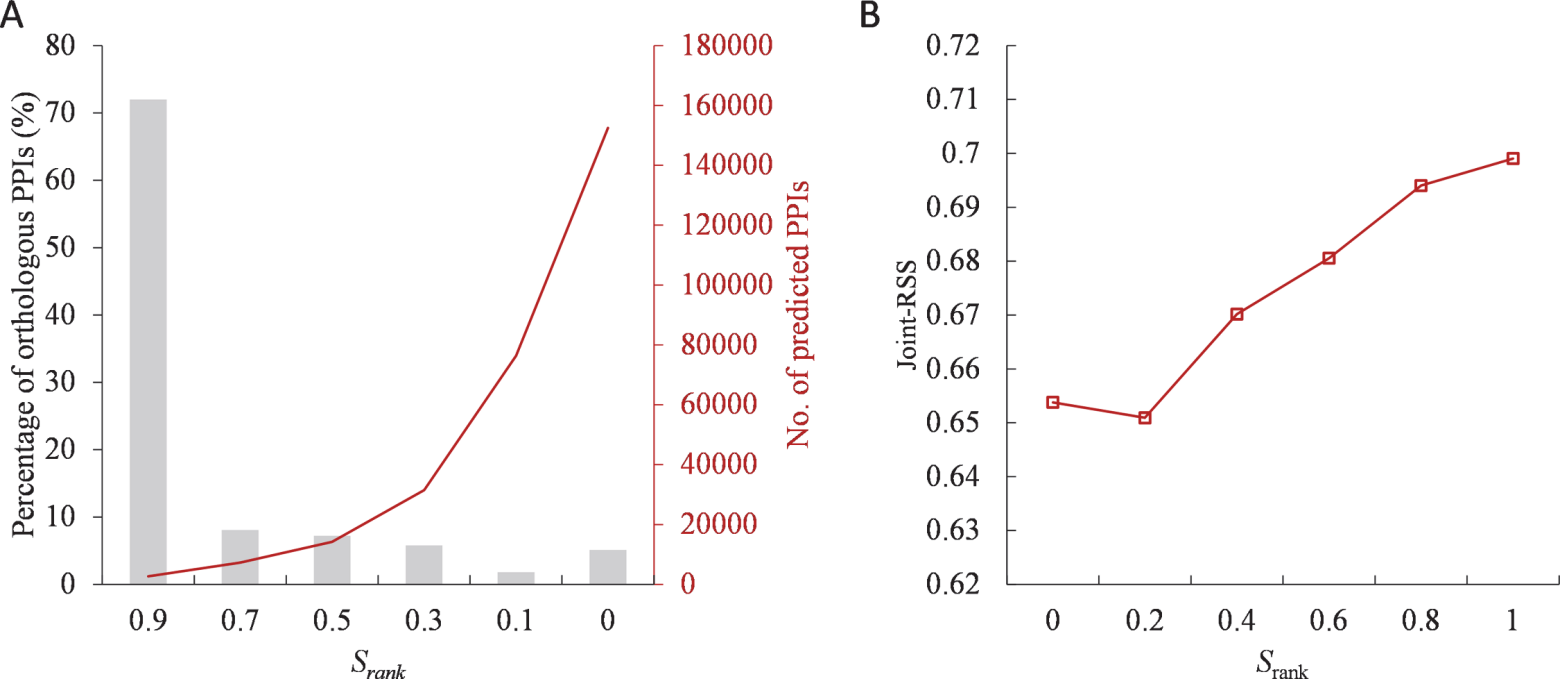
The relationships between normalized ranks (*S_rank_*) and orthologous interactions and joint-RSS scores on the YD set. (A) The distribution of orthologous interactions against normalized ranks. *S_rank_* of 72% orthologous interactions of a template PPI is more than 0.9. (B) The *S_rank_* scores of PPI candidates are correlated with their joint-*RSS* scores ($joint-RSS=\sqrt{RSS_{BP}\times RSS_{CC}}$).

Here, we use orthologous interactions and functional similarities to evaluate the reliability of predicted PPIs in the YD set. For the coverage of orthologous interactions, we collected orthologous proteins between the source and target organisms (i.e., *S. cerevisiae*) from the ENSEMBL database [30]. Among 106,825 PPI candidates, 2,639 PPIs are orthologous PPIs of 2,584 known PPIs. Among these 2,639 orthologous interactions, the *S_rank_* values of 72% (1,900 PPIs) and 89% (2,346 PPIs) of the orthologous interactions exceed 0.9 (*S_rank_* ≥ 0.9) and 0.5, respectively (Fig. 3A). Conversely, the sequence similarities (i.e., joint *E*-value) of these orthologous interactions are diverse (S5A Fig.). Furthermore, the distributions of orthologous interactions between the different numbers of PPI candidates with normalized rank score (*S_rank_*) and sequence similarities differ (S5B Fig.). The performance of *S_rank_* is significantly better than that of sequence similarities. These results imply that the sequence-based methods from a template PPI should consider few top-ranked homologous PPIs.

We then used the cellular component (CC) and biological process (BP) features of the Gene Ontology (GO) [31] to evaluate the functional similarity of predicted PPI candidates based on the YD set. Two interacting proteins often share similar cellular component and biological process. For a PPI candidate with pair proteins A and B, we used the *RSS* joint similarity ($joint-RSS=\sqrt{RSS_{BP}\times RSS_{CC}}$), to measure their functional similarity, where *RSS_BP_* and *RSS_CC_* are the relative specificity similarities of BP and CC, respectively. The *S_rank_* and multiple-strategy scores (*S*) of PPI candidates are highly correlated with their receptive joint-RSS score (Fig. 3B and S6 Fig.). These results imply that *S_rank_* is able to improve the PPI prediction accuracy and to reflect the biological functions. In addition, the top-ranked PPI candidates (*S_rank_* ≥ 0.9) are often orthologous interactions and share the functional similarity.

### Protein family size and length

**Figure 4:**
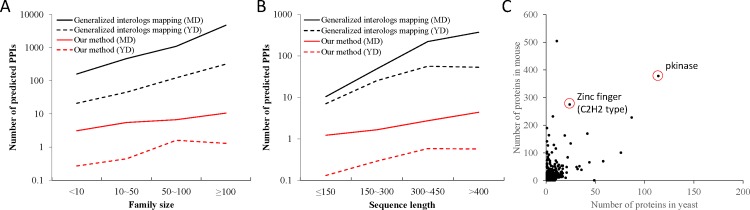
The relationship between the numbers of predicted PPIs with protein family sizes and protein lengths on YD and MD sets. The numbers of PPI candidates are highly correlated with (A) the number of homologous proteins (family size) and (B) protein sequence lengths of known PPI templates using generalized interologs mapping method (black lines) with joint sequence similarity (e.g., *E*-value ≤ 1 × 10^−40^). Conversely, the numbers of predicted PPIs of our method (red lines) are lightly influenced by the protein family sizes and lengths. (C) For a known PPI, the number of homologous proteins in mouse is significantly greater than the one of yeast. For example, the number of homologous proteins of a zinc-finger (PF00096) protein in mouse and yeast are 275 and 24, respectively.

Based on the interacting proteins (EPHB2 and ABL1) with the kinase domain, we inferred enormous numbers of homologous PPIs from the generalized interologs mapping method (Fig. 1C and S7 Fig.). To further investigate the criteria resulting in a great number of homologous PPIs, we analyzed the protein family size and protein length based on 10,047 and 75,105 known PPIs in the YD and MD sets, respectively (Fig. 4). Fig. 4A and 4B show that the numbers of predicted PPIs of our method (red lines) are lightly influenced by the protein family sizes and lengths. Conversely, the number of predicted PPI number of generalized interologs mapping method (black lines) significantly increases when the family sizes and protein length are enlarged.

The family sizes of two interacting proteins are highly correlated with the number of predicted PPI candidates using generalized interolog mapping because the proteins in a family often are homologous and share high sequence similarity (i.e., joint *E*-value ≤ 1 × 10^−40^ using BLASTP; Fig. 4A). For example, the generalized interolog mapping derived 6,838 PPI candidates in mouse for EPHB2-ABL1, the family sizes of which are 169 (EPHB2) and 257 (ABL1). Among these 6,838 candidates, our method can discard most false positive cases and retain 88 PPIs in which two PPIs were recorded in five public databases.

Fig. 4B shows the relationship between the average protein length and the numbers of candidates on the YD and MD sets. The number of PPI candidates using generalized interolog mapping increases as the average protein lengths of two interacting protein in both the YD and MD sets. Based on our best knowledge, the sequence similarities (i.e., BLASTP *E*-value) of homologous proteins are related to the lengths of query proteins. For example, the *E*-value between Abl1 (*M. musculus*, 1,123 amino acids) and ABL1 (*H. sapiens*, 1,130 amino acids) is 0. Conversely, the *E*-value between Snrpe (*M. musculus*, 92 amino acids) and SNRPE (*H. sapiens*, 92AAs) is 4 × 10^−49^.

The number of homologous proteins in mouse is much greater than that in yeast (Fig. 4C). According to 5,378 and 2,937 Pfam domains of 75,105 and 13,118 known PPIs in the MD and YD sets, respectively, 2,339 domains are conserved in both *M. musculus* and *S. cerevisiae*. We found that the number of proteins with conserved domains in *M. musculus* is significantly greater than that in *S. cerevisiae*. For example, the protein kinase domain (PF00069) is one of the largest families in both *M. musculus* and *S. cerevisiae*. There are 378 and 114 proteins with a protein kinase domain in mouse and yeast, respectively. Furthermore, the numbers of proteins with a zinc finger domain (PF00096) are 275 and 24 in mouse and yeast, respectively. The zinc finger domain is important for DNA binding, which is involved in gene regulation and translation [32]. These results indicate that the large family sizes of interacting proteins (e.g., kinase and zinc finger) result in enormous number of PPI candidates and often cause poor prediction accuracy.

### Conserved score *S_con_*

The conserved PPIs across multiple organisms are useful for reconstructing the PPI network of the target organism and aligning these multiple PPI networks. Here, we assume that the reliability of a predicted PPI depends on the evolutionary distance between the target and source organisms and then we used this evolutionary distance to infer the homologous PPIs in the target organism from multiple species. Because *H. sapiens* and *M. musculus* are Mammals and *D. melanogaster* is Pterygota based on InParanoid [22], the inferred PPIs in *M. musculus* from *H. sapiens* should be more reliable than that from *D. melanogaster*. Here, the evolutionary distances between *H. sapiens* and *D. melanogaster* to *M. musculus* are 0.765 and 0.235, respectively. For example, we can infer PPI Sos2-Grb2 in mouse from human (SOS2-GRB2) and fly (Sos-Drk; Fig. 1D).

The PPI FGFR2-FGF2 plays an important role in the regulation of cell survival, cell division, angiogenesis, cell differentiation and cell migration [33]. Because FGF2 is conserved in Euteleostomi according to annotation in the HomoloGene database, the PPI Fgfr2-Fgf2 in *M. musculus* can only be inferred from *H. sapiens* (Fig. 1D) and *S_con_* = 0.765. Based on the PPI FGFR2-FGF2 in human, our method can infer four reliable homologous PPIs, Fgfr1-Fgf2, Fgfr2-Fgf2, Fgfr3-Fgf2, and Fgfr4-Fgf2 in *M. musculus* (S3 Table). On the other hand, generalized interolog mapping uses the PPI shot-p115 in *D. melanogaster* to find the homologous PPI Plec-Uso1 in *M. musculus*. However, this Plec-Uso1interaction should be a false prediction [34,35], and our score *S_con_* = 0.235 for this interaction can discard this prediction (S4 Table).

### Reconstructed PPI network in *M. musculus*

We have evaluated the reliability of the homologous PPIs derived from our method and scoring function. Based on experimental PPIs and inferred homologous PPIs from generalized interologs mapping and our method, we reconstructed the mouse PPI networks and analyzed the properties and biological meanings (S8 and S10 Figs.). These three networks consist of 3,743 proteins and 6,855 PPIs using experimental PPIs, 19,326 proteins and 4,678,178 PPIs using predicted PPIs from generalized interologs mapping, and 12,921 proteins and 154,229 PPIs using PPIs predicted by our method. A biological network is often a scale-free network described as *P*(*k*) ~ *k* ^−γ^, in which the probability of a node with k links decreases as the node degree increases on a log–log plot (S8 Fig.). The degree exponent γ are 1.46, 0.92, and 1.50 in the PPI networks derived from experimental PPIs, generalized interologs mapping, and our method, respectively. Our network is consistent with the architecture (i.e., weak scale-free network properties) of some cellular networks [23,24]. A scale-free network typically has degree exponents 2 ≤ γ ≤ 3, but can also exist with 1 < γ < 2 [23,24]. Here, the γ value of the PPI network derived from generalized interologs mapping method is less than 1, so it is not considered as a scale-free network.

A module is a fundamental unit formed with highly connected proteins and often possesses specific biological functions. The interactions between modules are considered as the backbone of the cellular networks to regulate most biological processes [36,37]. We evaluated the behavior of modules in these three PPI networks in *M. musculus* using 216 pathways and 76 structural complexes collected from the KEGG database [38] (S5 Table). To observe connectivity (*C_t_*) of a KEGG module (M) which consists of a set (P) of proteins and a set (I) of protein-protein interactions (PPIs) in a PPI network, we quantified the connectivity by $C_{t}=m/C_{2}^{n}$ [39], where $C_{2}^{n}$ means *n* choose 2, *n* and *m* are the numbers of proteins and PPIs, respectively, in one M. For a KEGG module (M), we computed the connectivity values of module M (*C_M_*) and its one-layer-extended module (*C_M-extended_*). The one-layer-extended module of this module M includes a set (P⋃P’) of proteins and a set (I’) of PPIs, where P’ consists of the interacting proteins of each protein in set P; I’ consists of the PPIs of the proteins in the set P⋃P’. Here, we define the connectivity ratio of the module M as *C_M_* / *C_M-extended_* to evaluate its modularity in a PPI network. The high connectivity ratio of module M means that this module is high cohesion and low coupling in a network.

We utilized an example for describing the calculation of the connectivity ratio of a KEGG module (KEGG entry: mmu_M00148, succinate dehydrogenase complex) and its one-layer-extended module in the PPI networks derived from our method and experimental PPIs (S9 Fig.). The connectivity ratios are 4.5 (0.67/0.15) and 1.7 (0.5/0.29) for our method and experimental PPIs, respectively. The average connectivity ratios of 292 KEGG modules are 1.22, 1.97, and 4.69 in three networks, including experimental PPIs, generalized interologs mapping method, and our method, respectively (S10 Fig.). These results show that the reconstructed network (our method) has the highest connectivity ratio of 292 KEGG modules. Conversely, the reconstructed network using generalized interologs mapping method lost local compact because the network is near full connections.

### Example: EPHB2-ABL1 interaction

The Eph family of receptor tyrosine kinases (EPHB2) and Abl family of non-receptor tyrosine kinases (ABL1) participate in tissue morphogenesis in *H. sapiens* [40]. The EPHB2-ABL1 interaction can infer 6,838 PPIs in mouse using generalized interologs mapping because EPHB2 and ABL1 both possess a protein kinase domain (S7 Fig.). Among the 169 and 257 homologs of EPHB2 and ABL1, respectively, 169 (100%) and 245 (95%) proteins possess of the kinase domain and 6,838 protein pairs with joint *E*-values ≤ 1 × 10^−70^ were considered as PPI candidates. However, the interacting domains of EPHB2 and ABL1 are the type-A receptor 2 transmembrane (EphA2_TM) domain (PF14575: Ephrin) of EPHB2 and the SH2 domain (PF00017: SH2 domain) of ABL1 [40]. Furthermore, there are only 14 (8%) and 32 homologous proteins (12%) possessing the interacting domains of EPHB2 and ABL1, respectively. Among these 6,838 candidates, only 448 (6.6%) candidates maintain the interacting domain pairs (EphA2_TM and SH2).

Among these 6,838 candidates for the EPHB2-ABL1 interaction, our scoring method inferred 88 candidates meeting the criterion of *S* ≥ 2.3 and discarded the other PPIs with *S* < 2.3 and joint *E*-values ≤ 1 × 10^−70^ (e.g., Ephb2-Ptk2 and Src-Frk). These 88 predicted PPIs consist of the interacting domains EphA2_TM and SH2; conversely, most of the discarded PPIs do not possess the interacting domain nor have a low value of *S_rank_*. For example, for PPI Ephb2-Ptk2, Ptk2 is the homolog of ABL1, and it has three domains (Pkinase, FERM_M, and Focal_AT domains). However, Ptk2 does not have the SH2 domain. For the PPI Src-Frk, Src is the homolog of EPHB2, and it has four domains (PB014740, SH3, SH2, and Pkinase); however it does not have the EphA2_TM domain. This result shows that our method overcomes the disadvantages (the low coverage or prediction accuracy) of interolog and generalized interolog mapping.

### Example: Axon growth sub-network

**Figure 5:**
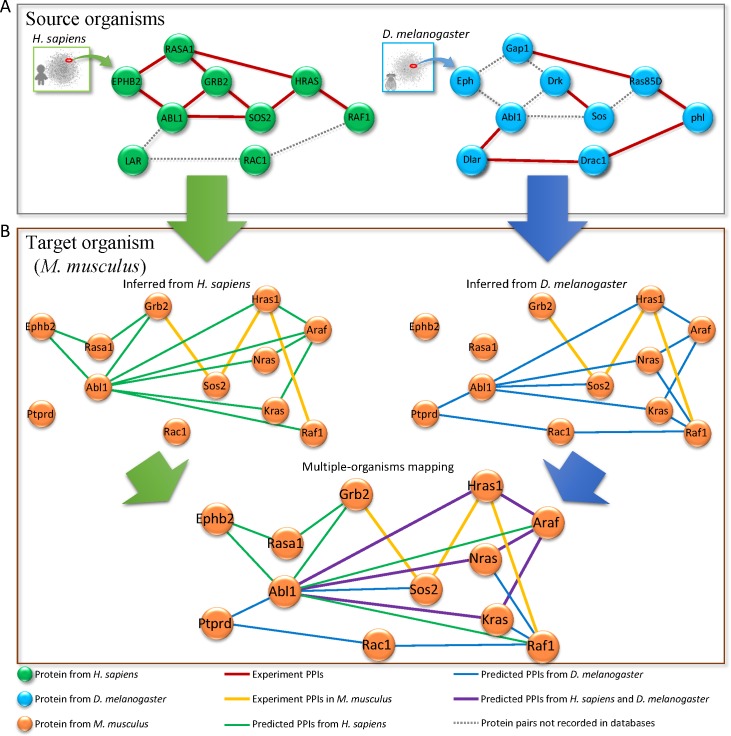
Inferred axon growth pathway in *M. musculus* from *H. sapiens* and *D. melanogaster*. The axon growth pathways of two source organisms, *H. sapiens* (green nodes) and *D. melanogaster* (blue nodes), use PPIs recorded in five public databases. The pathway of *M. musculus* (orange nodes) is based on the PPIs experiment PPIs (five public databases) and predicted PPIs from *H. sapiens* (light green edges) and *D. melanogaster* (light blue edges). The PPIs (purple edges) are inferred from two organisms. The dashed lines denote the predicted PPIs which are not recorded in these five databases.

EPHB2-ABL1 regulates the organization of the actin cytoskeleton in the developing nervous system and participates in axon growth signaling pathways [40]. Here, we used the downstream axon growth sub-network of EPHB2-ABL1 to describe the reconstructed sub-network of the target organism (Fig. 5A). The axon growth sub-networks in the source organisms (i.e., *H. sapiens* and *D. melanogaster*) were based on PPIs recorded in five databases. This sub-network consists of nine and six PPIs in *H. sapiens* and *D. melanogaster*, respectively. Three conserved PPIs (RASA1-HRAS, HRAS-RAF1, and GRB2-SOS2) are recorded in these two species. However, most of these experimental PPIs have not been evaluated in *M. musculus*. Three experimental PPIs (i.e. Grb2-Sos2, Sos2-Hras1 and Hras-Raf1) in *M. musculus* were recorded in public databases. Based on theses PPIs in the axon growth sub-networks of two source organisms, we inferred 22 PPIs to reconstruct a comprehensive axon growth sub-network in *M. musculus* (Fig. 5B). For example, Ephb2-Abl1 and Abl1-Grb2 cannot be inferred from *D. melanogaster*, but it can be inferred from *H. sapiens*. Conversely, Abl-Ptprd-Rac1-Raf1 cannot be inferred from *H. sapiens*, but they can be inferred from *D. melanogaster*. Moreover, two PPIs, Grb2-Sos2 and Hras1-Raf1, are derived from both *H. sapiens* (GRB2-SOS2, HRAS-RAF1) and *D. melanogaster* (Drk-Sos, Ras85D-phl). These two PPIs could be more reliable, and they were recorded in public databases. We believe that our method is useful for reconstructing PPI networks in interesting organisms.

### Sub-network spliceosome

**Figure 6:**
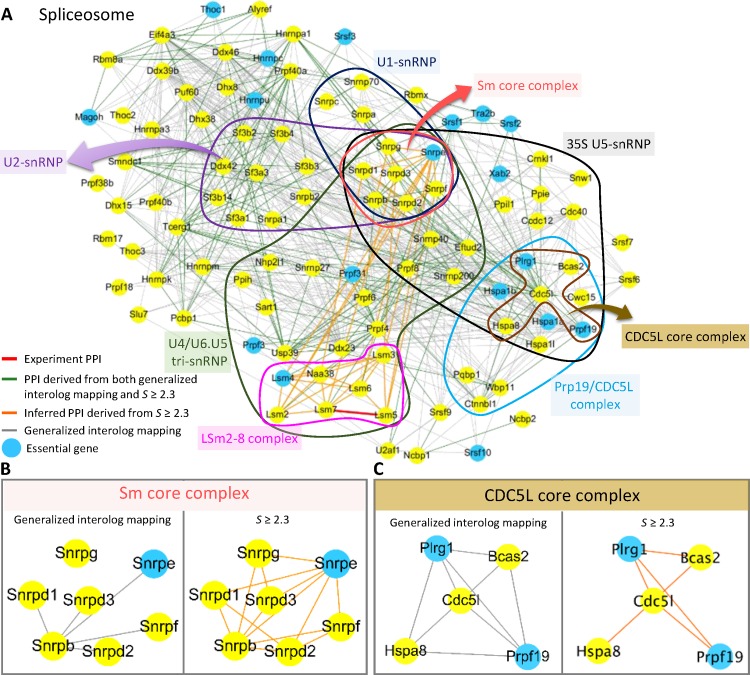
Reconstructed spliceosome sub-network of *M. musculus*. (A) The reconstructed spliceosome sub-network consists of including 99 proteins and 1,014 predicted PPIs, including 655 PPIs from only generalized interolog mapping methods (grey edges); 197 PPIs from only our method with *S* ≥ 2.3 (orange edges); and 162 PPIs from the overlap between generalized interolog mapping and our method (green edges). The sub-network consists of five structural complexes (i.e., U1-snRNP, U2-snRNP, U4/U6.U5 tri-snRNP, Prp19/CDC5L complex and 35S U5-snRNP) recorded in KEGG. Furthermore, three modules are recorded in the CORUM: Sm core, CDC5L core, and LSm2-8 complexes. The blue nodes are essential genes collected from the Mouse Genome Informatics database. (B) The two Sm core complexes are based on generalized interolog mapping (five PPIs) and our method with *S* ≥ 2.3 (14 PPIs). (C) The CDC5L core complex with five proteins and six PPIs based on our method (score *S* ≥ 2.3).

The spliceosome performs pre-mRNA splicing within the nucleus of eukaryotes, and it consists of five small nucleus robonclueoproteins (snRNPs) and numerous proteins [41]. Currently, there is only one annotated PPI (Lsm5-Lsm7) in *M. musculus*. Here, we reconstructed the spliceosome sub-network of *M. musculus*. This sub-network consists of 99 proteins and 1,014 predicted PPIs, including 655 PPIs from only generalized interolog mapping methods (grey edges in Fig. 6A); 197 PPIs from only our method with *S* ≥ 2.3 (orange edges in Fig. 6A); and 162 PPIs from the overlap between generalized interolog mapping and our method (green edges in Fig. 6A). Based on the KEGG spliceosome pathway, this sub-network primarily consists of five modules: U1-snRNP, U2-snRNP, U4/U6.U5 tri-snRNP, Prp19/CDC5L complex and 35S U5-snRNP. U1-snRNP is important for pre-mRNA splicing in both yeast and mammalian systems. The RNA component of U1-snRNP and U1-snRNA performs base pairing with pre-mRNA 5′ splice sites [42]. The Prp19/CDC5L complex plays a central role during catalytic activation of the spliceosome, and Prp19 and its related proteins are major components of the spliceosome’s catalytic core RNP [43]. In addition, three core modules are recorded in CORUM: Sm core complex, CDC5L core complex, and LSm2-8 complex [44]. The blue nodes are the essential genes collected from Mouse Genome Informatics (MGI) database [45].

The Sm complex plays an essential role in the formation of snRNPs by binding to small nuclear RNAs [29], and no PPIs are currently recorded in databases. Based on the predicted PPIs from generalized interolog mapping (five PPIs) and our method (14 PPIs score *S* ≥ 2.3, S6 Table), we reconstructed the Sm sub-network Fig. 6B. Our method provides additional nine PPIs, including Snrpe-Snrpg, Snrpe-Snrpf, Snrpe-Snrpd1, Snrpe-Snprd2, Snrpg-Snrpd3, Snrpg-Snrpb, Snrpe-Snrpd3, Snrpd1-Snrpd2, and Snrpf-Snrpd2, the joint sequence similarities of which exceed 1 × 10^−70^ because the sequence lengths of these proteins are less than 150 amino acids. For instance, the sequence lengths of Snrpe and Snrpg are 92 and 76 amino acids in mouse, respectively. The sequence similarities between mouse and human homologous proteins SNRPE and SNRPG are 8 × 10^−61^ and 1 × 10^−58^, respectively. After considering rank (*S_rank_* = 1) and consensus across multiple organisms (*S_con_* = 0.765 because the Sm complex can only be derived from *H. sapiens*), the PPI Snrpe-Snrpg (S = 2.639) can be predicted by our method. These six PPIs should be essential for the Sm complex [29,46].

The cell division cycle 5-like (CDC5L) complex is essential for spliceosome assembly and catalysis [47]. We reconstructed the CDC5L sub-network, which consists of five proteins and six PPIs (Fig. 6C). One of the six PPIs is essential proteins pair, pleiotropic regulator 1 (Plrg1) and pre-mRNA-processing factor 19 (Prpf19) contain the WD40 domain (PF00400) which consist of highly conserved repeating units usually ending with Trp-Asp (WD), and they are found in all eukaryotes but not in prokaryotes [48]. According to crystal structure (PDB ID: 4LG8), the interacting domains are WD40-WD40, and previous reports support the interaction PLRG1-PRPF19 in *H. sapiens* [43,49,50]. These evidences show that the PPI Plrg10-Prpf19 is a reliable interaction.

## Conclusions

We propose a multiple-strategy scoring method, combining sequence similarities, normalized ranks, and conservation scores across multiple organisms, to identify reliable PPIs for reconstructing biological networks using homologous PPIs from complete genomic database. Our multiple-strategy score is able to avoid the disadvantages (e.g. high false-positive rate) of sequence-based homologous mapping methods to predict reliable genome-scale PPIs for an interesting organism. We found that the top-ranked and conserved PPIs are often orthologous/essential interactions and share the functional similarity. Based on our predicted PPIs, we can reconstruct a comprehensive PPI network of a target organism and this network is a scale-free network and can reflect the biological functions and module properties (such as sharing similar biological processes and cellular component; network topology). We believe that our method and scoring function are useful for inferring genome-wide PPIs as well as reconstructing and aligning multiple PPI networks for multiple organisms.

## Supporting Information

S1 Table Summary of parameter tests with liner and non-liner combinations. (DOCX)

S2 Table The homologous PPIs derived from SNRPG-SNRPE and SNRPE-SNRPF. (DOCX)

S3 Table The homologous PPIs derived from FGFR2-FGF2. (DOCX)

S4 Table The homologous PPIs derived from shot-p115. (DOCX)

S5 Table 216 pathways and 76 structural complexes derived from KEGG. (DOCX)

S6 Table 14 PPIs derived from our method in the Sm complex. (DOCX)

S1 Fig The parameter test of weights (i.e. *w_1_*, *w_2_* and *w_3_*) for the liner combination of *S_sim_*, *S_rank_*, and *S_con_* on the YD set. The *w_1_*, *w_2_*, and *w_3_* values are tested by various values ranging from 0 to 1. Finally, the *w_1_*, *w_2_*, and *w_3_* are set to 1.
(TIF)

S2 FigThe values of α, β and γ for calculating the RSS of mitochondrial genome maintenance (GO:0000002) and mitochondrial fragmentation involved in apoptotic process (GO:0043653).The RSS of two GO terms, *t_i_* and *t_j_*, is calculated by: $\mathrm{RSS}(t_{i},t_{j})=\frac{{\mathrm{maxD}}^{\mathrm{GO}}}{{\mathrm{maxD}}^{\mathrm{GO}}+\gamma }\times \frac{\alpha }{\alpha +\beta }$, where *maxD* ^GO^ is the maximum depth from the root term of the GO to the leaf terms (i.e., *maxD^BP^* = 20 and *maxD^CC^* = 18 based on data version: releases 2014-05-29); α is the depth from the root term to most recent common ancestor (MRCA) of *t_i_* and *t_j_*; β is the max(*DL_i_*, *DL_j_*), where *DL_i_* and *DL_j_* are the minimum depths from *t_i_* to its leaf terms and *t_j_* to its leaf terms, respectively; γ is sum of distances between MRCA to *t_i_* and MRCA to *t_j_*. Therefore, the RSS (GO:0000002, GO:0043653) is (20/(20+3)) × (4/(4+1)) = 0.696.(TIF)

S3 Fig Performance of six scoring combinations on the MD set. (A) The ROC curves of six scoring combinations, including normalized joint sequence similarity (*S_sim_*), normalized rank (*S_rank_*), conserved score (*S_con_*), *S_sim_* + *S_rank_*, *S_sim_* + *S_con_*, and *S_sim_* + *S_rank_* + *S_con_*, on the MD set for the target organism, *M. musculus*. (B) The relationship between the number of true-positive cases and the precision of these six scoring combinations. Among these six combinations, *S_sim_* + *S_rank_* + *S_con_* is the best.
(TIF)

S4 Fig The relationship between the number of candidates and prediction accuracies in the MD and YD sets. The prediction accuracy of the generalized interologs mapping decreases as the number of PPI candidates increases in both the YD and MD sets. The number of candidates derived from known PPIs increases from *S. cerevisiae* to *M. musculus*.
(TIF)

S5 Fig Distributions of orthologous protein-protein interactions in the YD set. The orthologous interaction means an interacting orthologs protein pair of the template PPI in the source organisms. (A) The distribution of orthologous interactions against the *E*-value using BLASTP. (B) The distributions of orthologous interactions under different numbers of predicted PPIs derived from the normalized rank (*S_rank_*) and sequence similarities (i.e., joint *E*-value).
(TIF)

S6 Fig The relationship between the multiple-strategy score (*S*) and Joint-RSS score on the YD set. The score (*S*) is highly correlated with Joint-RSS score.
(TIF)

S7 Fig The generalized interologs mapping with ranking in M. musculus derived from EPHB2-ABL1 in *H. sapiens*. The generalized interologs mapping derived from EPHB2-ABL1 includes 6,838 PPIs. The major domain of both EPHB2 and ABL1 is a kinase domain (blue color). Most homologous proteins of EPHB2 and ABL1 derived from generalized interologs mapping are the kinase domains (100% and 95%, respectively). However, the interacting domains of EPHB2 and ABL1 are the EphA2_TM (green color) and SH2 domain (brown color), respectively. There are only 488 PPIs (6.6%) that retain the interacting domain pairs.
(TIF)

S8 Fig Node degree distributions of mouse PPI networks derived from (A) experimental PPIs, (B) generalized interologs mapping, and (C) our method. PPI networks derived from experimental PPIs and our method are consistent with the weak scale-free network architectures of some cellular networks.
(TIF)

S9 Fig The connectivity of succinate dehydrogenase complex (KEGG entry: mmu_M00148) and one-layer-extended module on the PPI networks derived from our methods and experimental PPIs. The succinate dehydrogenase complexes have four and three PPIs derived from (A) our methods and (B) experimental PPIs, respectively. The one-layer-extended modules have 49 and 6 PPIs derived from (C) our methods and (D) experimental PPIs, respectively.
(TIF)

S10 Fig Connectivity ratio of the 292 KEGG modules and one-layer-extended modules in PPI networks of *M. musculus* derived from experimental PPIs, generalized interologs mapping, and our method. (A) Connectivity ratio of 216 KEGG pathways and one-layer-extended pathways. (B) Connectivity ratio of 76 KEGG complexes and one-layer-extended complexes. The pathways and modules prefer to have highly connected proteins and local compactness (e.g. highly connectivity ratios between modules and one-layer-extended modules) on the networks derived from our methods and experimental PPIs.
(TIF)
