# Supplementary material for: Reconstructing Genome-Wide Protein–Protein Interaction Networks Using Multiple Strategies with Homologous Mapping
Source: PLoS One. 2015 Jan 20;10(1):e0116347. doi: 10.1371/journal.pone.0116347 (PMC4300222; doi:10.1371/journal.pone.0116347)
Supplement: S1 Table — (DOCX) [file pone.0116347.s001.docx]

**Table S1. Summary of parameter tests with liner and non-liner combinations**

| Parameter test | *w_1_* | *w_2_* | *w_3_* | Area under the Curve  of ROC (AUC) |
| --- | --- | --- | --- | --- |
| Liner combinations  *w_1_S_sim_* + *w_2_S_rank_* + *w_3_S_con_* | 1 | 1 | 1 | 0.821 |
|  | 1 | 1 | 0 | 0.797 |
|  | 1 | 0 | 1 | 0.685 |
|  | 1 | 0 | 0 | 0.635 |
|  | 0 | 1 | 0 | 0.782 |
|  | 0 | 0 | 1 | 0.574 |
| Weight derived from AUC of  using AUC area of the single term as the weight  (i.e., (1,0,0), (0,1,0), and (0,0,1))^a^ | 0.32 | 0.39 | 0.29 | 0.818 |
| Non-liner combinations  *S_sim_* × *S_rank_* × *S_con_* | 1 | 1 | 1 | 0.795 |

^a^The *w_1_*, *w_2_*, *w_3_* are computed as

*w_1_* = 0.635/ (0.635+0.782+0.574), *w_2_* = 0.782/ (0.635+0.782+0.574), and

*w_3_* = 0.574/ (0.635+0.782+0.574)
