# Supplementary material for: Reconstructing Genome-Wide Protein–Protein Interaction Networks Using Multiple Strategies with Homologous Mapping
Source: PLoS One. 2015 Jan 20;10(1):e0116347. doi: 10.1371/journal.pone.0116347 (PMC4300222; doi:10.1371/journal.pone.0116347)
Supplement: S2 Table — (DOCX) [file pone.0116347.s002.docx]

**Table S2. The homologous PPIs derived from SNRPG-SNRPE and SNRPE-SNRPF**

| Protein  (A') | Gene name | Protein  (B) | Gene name | *E*-value (A') | SI^*^ (A') | *E*-value (B') | SI^*^ (B') | Joint *E*-value | *S_sim_* | *S_rank_* | *S_con_* | *S* |
| --- | --- | --- | --- | --- | --- | --- | --- | --- | --- | --- | --- | --- |
| P62309 | Snrpg | P62305 | Snrpe | 47.398 | 100 | 60 | 100 | 53.699 | 0.874 | 1 | 0.765 | 2.639 |
| P62305 | Snrpe | P62307 | Snrpf | 60 | 100 | 54.398 | 100 | 57.199 | 0.883 | 1 | 0.765 | 2.648 |

^*^Sequence identity (SI)
