# Supplementary material for: Reconstructing Genome-Wide Protein–Protein Interaction Networks Using Multiple Strategies with Homologous Mapping
Source: PLoS One. 2015 Jan 20;10(1):e0116347. doi: 10.1371/journal.pone.0116347 (PMC4300222; doi:10.1371/journal.pone.0116347)
Supplement: S3 Table — (DOCX) [file pone.0116347.s003.docx]

**Table S3. The homologous PPIs derived from FGFR2-FGF2**

| Protein  (A') | Gene name | Protein (B') | Gene name | *E*-value (A') | SI^*^ (A') | *E*-value (B') | SI^*^ (B') | Rank | *S_sim_* | *S_rank_* | *S_con_* | *S* |
| --- | --- | --- | --- | --- | --- | --- | --- | --- | --- | --- | --- | --- |
| P21803 | Fgfr2 | P15655 | Fgf2 | 180 | 97 | 103 | 94.8 | 1 | 0.786 | 1 | 0.765 | 2.551 |
| P16092 | Fgfr1 | P15655 | Fgf2 | 180 | 71.8 | 103 | 94.8 | 2 | 0.786 | 0.877 | 0.765 | 2.428 |
| Q61851 | Fgfr3 | P15655 | Fgf2 | 180 | 68.1 | 103 | 94.8 | 3 | 0.786 | 0.805 | 0.765 | 2.356 |
| Q03142 | Fgfr4 | P15655 | Fgf2 | 180 | 57.7 | 103 | 94.8 | 4 | 0.786 | 0.754 | 0.765 | 2.305 |
| P21803 | Fgfr2 | P61148 | Fgf1 | 180 | 97 | 44.7 | 53.5 | 5 | 0.624 | 0.714 | 0.765 | 2.103 |
| P16092 | Fgfr1 | P61148 | Fgf1 | 180 | 71.8 | 44.7 | 53.5 | 6 | 0.624 | 0.682 | 0.765 | 2.071 |
| Q61851 | Fgfr3 | P61148 | Fgf1 | 180 | 68.1 | 44.7 | 53.5 | 7 | 0.624 | 0.654 | 0.765 | 2.043 |
| Q03142 | Fgfr4 | P61148 | Fgf1 | 180 | 57.7 | 44.7 | 53.5 | 8 | 0.624 | 0.631 | 0.765 | 2.02 |
| P21803 | Fgfr2 | P21658 | Fgf6 | 180 | 97 | 22 | 43.7 | 9 | 0.561 | 0.61 | 0.765 | 1.936 |
| P16092 | Fgfr1 | P21658 | Fgf6 | 180 | 71.8 | 22 | 43.7 | 10 | 0.561 | 0.591 | 0.765 | 1.917 |
| Q61851 | Fgfr3 | P21658 | Fgf6 | 180 | 68.1 | 22 | 43.7 | 11 | 0.561 | 0.574 | 0.765 | 1.9 |
| Q03142 | Fgfr4 | P21658 | Fgf6 | 180 | 57.7 | 22 | 43.7 | 12 | 0.561 | 0.559 | 0.765 | 1.885 |
| P35546 | Ret | P15655 | Fgf2 | 98.2 | 50 | 103 | 94.8 | 13 | 0.559 | 0.545 | 0.765 | 1.869 |
| P35918 | Kdr | P15655 | Fgf2 | 97.5 | 35.2 | 103 | 94.8 | 14 | 0.557 | 0.531 | 0.765 | 1.853 |
| P21803 | Fgfr2 | P15656 | Fgf5 | 180 | 97 | 19.7 | 43 | 15 | 0.555 | 0.519 | 0.765 | 1.839 |
| P21803 | Fgfr2 | P05524 | Fgf3 | 180 | 97 | 19.7 | 41.6 | 16 | 0.555 | 0.508 | 0.765 | 1.828 |
| P16092 | Fgfr1 | P15656 | Fgf5 | 180 | 71.8 | 19.7 | 43 | 17 | 0.555 | 0.497 | 0.765 | 1.817 |
| P16092 | Fgfr1 | P05524 | Fgf3 | 180 | 71.8 | 19.7 | 41.6 | 18 | 0.555 | 0.487 | 0.765 | 1.807 |
| Q61851 | Fgfr3 | P15656 | Fgf5 | 180 | 68.1 | 19.7 | 43 | 19 | 0.555 | 0.477 | 0.765 | 1.797 |
| Q61851 | Fgfr3 | P05524 | Fgf3 | 180 | 68.1 | 19.7 | 41.6 | 20 | 0.555 | 0.468 | 0.765 | 1.788 |
| Q03142 | Fgfr4 | P15656 | Fgf5 | 180 | 57.7 | 19.7 | 43 | 21 | 0.555 | 0.459 | 0.765 | 1.779 |
| Q03142 | Fgfr4 | P05524 | Fgf3 | 180 | 57.7 | 19.7 | 41.6 | 22 | 0.555 | 0.451 | 0.765 | 1.771 |
| P21803 | Fgfr2 | P11403 | Fgf4 | 180 | 97 | 19.1 | 42 | 23 | 0.553 | 0.443 | 0.765 | 1.761 |
| P16092 | Fgfr1 | P11403 | Fgf4 | 180 | 71.8 | 19.1 | 42 | 24 | 0.553 | 0.436 | 0.765 | 1.754 |
| Q61851 | Fgfr3 | P11403 | Fgf4 | 180 | 68.1 | 19.1 | 42 | 25 | 0.553 | 0.428 | 0.765 | 1.746 |
| Q03142 | Fgfr4 | P11403 | Fgf4 | 180 | 57.7 | 19.1 | 42 | 26 | 0.553 | 0.421 | 0.765 | 1.739 |
| P21803 | Fgfr2 | Q9ESL9 | Fgf20 | 180 | 97 | 16.5 | 37.5 | 27 | 0.546 | 0.415 | 0.765 | 1.726 |
| P16092 | Fgfr1 | Q9ESL9 | Fgf20 | 180 | 71.8 | 16.5 | 37.5 | 28 | 0.546 | 0.408 | 0.765 | 1.719 |
| Q61851 | Fgfr3 | Q9ESL9 | Fgf20 | 180 | 68.1 | 16.5 | 37.5 | 29 | 0.546 | 0.402 | 0.765 | 1.713 |
| Q03142 | Fgfr4 | Q9ESL9 | Fgf20 | 180 | 57.7 | 16.5 | 37.5 | 30 | 0.546 | 0.396 | 0.765 | 1.707 |
| P21803 | Fgfr2 | P54130 | Fgf9 | 180 | 97 | 16.3 | 39.4 | 31 | 0.545 | 0.39 | 0.765 | 1.7 |
| P16092 | Fgfr1 | P54130 | Fgf9 | 180 | 71.8 | 16.3 | 39.4 | 32 | 0.545 | 0.385 | 0.765 | 1.695 |
| Q61851 | Fgfr3 | P54130 | Fgf9 | 180 | 68.1 | 16.3 | 39.4 | 33 | 0.545 | 0.379 | 0.765 | 1.689 |
| Q03142 | Fgfr4 | P54130 | Fgf9 | 180 | 57.7 | 16.3 | 39.4 | 34 | 0.545 | 0.374 | 0.765 | 1.684 |
| P21803 | Fgfr2 | Q9ESL8 | Fgf16 | 180 | 97 | 15.7 | 40.2 | 35 | 0.544 | 0.369 | 0.765 | 1.678 |
| P16092 | Fgfr1 | Q9ESL8 | Fgf16 | 180 | 71.8 | 15.7 | 40.2 | 36 | 0.544 | 0.364 | 0.765 | 1.673 |
| Q61851 | Fgfr3 | Q9ESL8 | Fgf16 | 180 | 68.1 | 15.7 | 40.2 | 37 | 0.544 | 0.359 | 0.765 | 1.668 |
| Q03142 | Fgfr4 | Q9ESL8 | Fgf16 | 180 | 57.7 | 15.7 | 40.2 | 38 | 0.544 | 0.354 | 0.765 | 1.663 |
| P21803 | Fgfr2 | O35565 | Fgf10 | 180 | 97 | 14.4 | 36.4 | 39 | 0.54 | 0.349 | 0.765 | 1.654 |
| P16092 | Fgfr1 | O35565 | Fgf10 | 180 | 71.8 | 14.4 | 36.4 | 40 | 0.54 | 0.345 | 0.765 | 1.65 |
| Q61851 | Fgfr3 | O35565 | Fgf10 | 180 | 68.1 | 14.4 | 36.4 | 41 | 0.54 | 0.341 | 0.765 | 1.646 |
| Q03142 | Fgfr4 | O35565 | Fgf10 | 180 | 57.7 | 14.4 | 36.4 | 42 | 0.54 | 0.336 | 0.765 | 1.641 |
| P21803 | Fgfr2 | P70379 | Fgf14 | 180 | 97 | 12.7 | 34.9 | 43 | 0.535 | 0.332 | 0.765 | 1.632 |
| P16092 | Fgfr1 | P70379 | Fgf14 | 180 | 71.8 | 12.7 | 34.9 | 44 | 0.535 | 0.328 | 0.765 | 1.628 |
| Q61851 | Fgfr3 | P70379 | Fgf14 | 180 | 68.1 | 12.7 | 34.9 | 45 | 0.535 | 0.324 | 0.765 | 1.624 |
| Q03142 | Fgfr4 | P70379 | Fgf14 | 180 | 57.7 | 12.7 | 34.9 | 46 | 0.535 | 0.32 | 0.765 | 1.62 |
| P21803 | Fgfr2 | Q9ESS2 | Fgf22 | 180 | 97 | 12.3 | 34.6 | 47 | 0.534 | 0.316 | 0.765 | 1.615 |
| P21803 | Fgfr2 | P61329 | Fgf12 | 180 | 97 | 12.3 | 32.5 | 48 | 0.534 | 0.313 | 0.765 | 1.612 |
| P16092 | Fgfr1 | Q9ESS2 | Fgf22 | 180 | 71.8 | 12.3 | 34.6 | 49 | 0.534 | 0.309 | 0.765 | 1.608 |
| Q61851 | Fgfr3 | Q9ESS2 | Fgf22 | 180 | 68.1 | 12.3 | 34.6 | 50 | 0.534 | 0.305 | 0.765 | 1.604 |
| P16092 | Fgfr1 | P61329 | Fgf12 | 180 | 71.8 | 12.3 | 32.5 | 51 | 0.534 | 0.302 | 0.765 | 1.601 |
| Q61851 | Fgfr3 | P61329 | Fgf12 | 180 | 68.1 | 12.3 | 32.5 | 52 | 0.534 | 0.298 | 0.765 | 1.597 |
| Q03142 | Fgfr4 | Q9ESS2 | Fgf22 | 180 | 57.7 | 12.3 | 34.6 | 53 | 0.534 | 0.295 | 0.765 | 1.594 |
| Q03142 | Fgfr4 | P61329 | Fgf12 | 180 | 57.7 | 12.3 | 32.5 | 54 | 0.534 | 0.292 | 0.765 | 1.591 |
| P21803 | Fgfr2 | P36363 | Fgf7 | 180 | 97 | 12 | 37.8 | 55 | 0.533 | 0.288 | 0.765 | 1.586 |
| P16092 | Fgfr1 | P36363 | Fgf7 | 180 | 71.8 | 12 | 37.8 | 56 | 0.533 | 0.285 | 0.765 | 1.583 |
| Q61851 | Fgfr3 | P36363 | Fgf7 | 180 | 68.1 | 12 | 37.8 | 57 | 0.533 | 0.282 | 0.765 | 1.58 |
| Q03142 | Fgfr4 | P36363 | Fgf7 | 180 | 57.7 | 12 | 37.8 | 58 | 0.533 | 0.279 | 0.765 | 1.577 |
| P35969 | Flt1 | P15655 | Fgf2 | 87.7 | 46.6 | 103 | 94.8 | 59 | 0.53 | 0.276 | 0.765 | 1.571 |
| P21803 | Fgfr2 | P70377 | Fgf13 | 180 | 97 | 10.4 | 32.5 | 60 | 0.529 | 0.273 | 0.765 | 1.567 |
| P16092 | Fgfr1 | P70377 | Fgf13 | 180 | 71.8 | 10.4 | 32.5 | 61 | 0.529 | 0.27 | 0.765 | 1.564 |
| Q61851 | Fgfr3 | P70377 | Fgf13 | 180 | 68.1 | 10.4 | 32.5 | 62 | 0.529 | 0.267 | 0.765 | 1.561 |
| Q03142 | Fgfr4 | P70377 | Fgf13 | 180 | 57.7 | 10.4 | 32.5 | 63 | 0.529 | 0.264 | 0.765 | 1.558 |
| P21803 | Fgfr2 | P37237 | Fgf8 | 180 | 97 | 10.3 | 30.6 | 64 | 0.529 | 0.261 | 0.765 | 1.555 |
| P16092 | Fgfr1 | P37237 | Fgf8 | 180 | 71.8 | 10.3 | 30.6 | 65 | 0.529 | 0.259 | 0.765 | 1.553 |
| Q61851 | Fgfr3 | P37237 | Fgf8 | 180 | 68.1 | 10.3 | 30.6 | 66 | 0.529 | 0.256 | 0.765 | 1.55 |
| Q03142 | Fgfr4 | P37237 | Fgf8 | 180 | 57.7 | 10.3 | 30.6 | 67 | 0.529 | 0.253 | 0.765 | 1.547 |
| P35917 | Flt4 | P15655 | Fgf2 | 87.1 | 34.6 | 103 | 94.8 | 68 | 0.528 | 0.251 | 0.765 | 1.544 |
| P05532 | Kit | P15655 | Fgf2 | 78.5 | 42.4 | 103 | 94.8 | 69 | 0.504 | 0.248 | 0.765 | 1.517 |
| P09581 | Csf1r | P15655 | Fgf2 | 76.3 | 42.5 | 103 | 94.8 | 70 | 0.498 | 0.246 | 0.765 | 1.509 |
| Q02858 | Tek | P15655 | Fgf2 | 67.7 | 46.2 | 103 | 94.8 | 71 | 0.474 | 0.243 | 0.765 | 1.482 |
| Q06806 | Tie1 | P15655 | Fgf2 | 67.7 | 45.4 | 103 | 94.8 | 72 | 0.474 | 0.241 | 0.765 | 1.48 |
| Q60751 | Igf1r | P15655 | Fgf2 | 60.1 | 40.1 | 103 | 94.8 | 73 | 0.453 | 0.238 | 0.765 | 1.456 |
| Q6VNS1 | Ntrk3 | P15655 | Fgf2 | 59.7 | 27.1 | 103 | 94.8 | 74 | 0.452 | 0.236 | 0.765 | 1.453 |
| Q8BKG3 | Ptk7 | P15655 | Fgf2 | 58.7 | 27.8 | 103 | 94.8 | 75 | 0.449 | 0.233 | 0.765 | 1.447 |
| P00520 | Abl1 | P15655 | Fgf2 | 58.2 | 39.5 | 103 | 94.8 | 76 | 0.448 | 0.231 | 0.765 | 1.444 |
| Q4JIM5 | Abl2 | P15655 | Fgf2 | 58.1 | 39.9 | 103 | 94.8 | 77 | 0.447 | 0.229 | 0.765 | 1.441 |
| Q9WTL4 | Insrr | P15655 | Fgf2 | 57.7 | 37.8 | 103 | 94.8 | 78 | 0.446 | 0.226 | 0.765 | 1.437 |
| Q61006 | Musk | P15655 | Fgf2 | 56.5 | 40.2 | 103 | 94.8 | 79 | 0.443 | 0.224 | 0.765 | 1.432 |
| P15208 | Insr | P15655 | Fgf2 | 56.3 | 39.6 | 103 | 94.8 | 80 | 0.443 | 0.222 | 0.765 | 1.43 |
| Q62270 | Srms | P15655 | Fgf2 | 56.1 | 38.2 | 103 | 94.8 | 81 | 0.442 | 0.22 | 0.765 | 1.427 |
| P70451 | Fer | P15655 | Fgf2 | 55.5 | 38.3 | 103 | 94.8 | 82 | 0.44 | 0.217 | 0.765 | 1.422 |
| P41242 | Matk | P15655 | Fgf2 | 55.2 | 39.2 | 103 | 94.8 | 83 | 0.44 | 0.215 | 0.765 | 1.42 |
| P15209 | Ntrk2 | P15655 | Fgf2 | 55.2 | 27.1 | 103 | 94.8 | 84 | 0.44 | 0.213 | 0.765 | 1.418 |
| Q3UFB7 | Ntrk1 | P15655 | Fgf2 | 54.7 | 36.9 | 103 | 94.8 | 85 | 0.438 | 0.211 | 0.765 | 1.414 |
| P41241 | Csk | P15655 | Fgf2 | 53.5 | 37.2 | 103 | 94.8 | 86 | 0.435 | 0.209 | 0.765 | 1.409 |
| Q00993 | Axl | P15655 | Fgf2 | 53.4 | 37.9 | 103 | 94.8 | 87 | 0.434 | 0.207 | 0.765 | 1.406 |
| P97793 | Alk | P15655 | Fgf2 | 53.3 | 37 | 103 | 94.8 | 88 | 0.434 | 0.205 | 0.765 | 1.404 |
| Q64434 | Ptk6 | P15655 | Fgf2 | 53 | 42 | 103 | 94.8 | 89 | 0.433 | 0.203 | 0.765 | 1.401 |
| Q00342 | Flt3 | P15655 | Fgf2 | 53 | 36.4 | 103 | 94.8 | 90 | 0.433 | 0.201 | 0.765 | 1.399 |
| Q60805 | Mertk | P15655 | Fgf2 | 52.7 | 33.7 | 103 | 94.8 | 91 | 0.432 | 0.199 | 0.765 | 1.396 |
| Q922K9 | Frk | P15655 | Fgf2 | 52.4 | 40.3 | 103 | 94.8 | 92 | 0.432 | 0.197 | 0.765 | 1.394 |
| P55144 | Tyro3 | P15655 | Fgf2 | 52.4 | 37.2 | 103 | 94.8 | 93 | 0.432 | 0.195 | 0.765 | 1.392 |
| Q78DX7 | Ros1 | P15655 | Fgf2 | 52.3 | 40.9 | 103 | 94.8 | 94 | 0.431 | 0.193 | 0.765 | 1.389 |
| P08923 | Ltk | P15655 | Fgf2 | 52 | 36.8 | 103 | 94.8 | 95 | 0.431 | 0.191 | 0.765 | 1.387 |
| P16879 | Fes | P15655 | Fgf2 | 51.7 | 35.3 | 103 | 94.8 | 96 | 0.43 | 0.189 | 0.765 | 1.384 |
| Q9Z138 | Ror2 | P15655 | Fgf2 | 50.7 | 32.1 | 103 | 94.8 | 97 | 0.427 | 0.188 | 0.765 | 1.38 |
| P26618 | Pdgfra | P15655 | Fgf2 | 50.1 | 53.9 | 103 | 94.8 | 98 | 0.425 | 0.186 | 0.765 | 1.376 |
| P34152 | Ptk2 | P15655 | Fgf2 | 50 | 34.2 | 103 | 94.8 | 99 | 0.425 | 0.184 | 0.765 | 1.374 |
| Q9Z139 | Ror1 | P15655 | Fgf2 | 49.7 | 35.1 | 103 | 94.8 | 100 | 0.424 | 0.182 | 0.765 | 1.371 |
| P06240 | Lck | P15655 | Fgf2 | 49.2 | 38.3 | 103 | 94.8 | 101 | 0.423 | 0.18 | 0.765 | 1.368 |
| Q62190 | Mst1r | P15655 | Fgf2 | 49 | 34 | 103 | 94.8 | 102 | 0.422 | 0.179 | 0.765 | 1.366 |
| P97504 | Bmx | P15655 | Fgf2 | 48.5 | 35.7 | 103 | 94.8 | 103 | 0.421 | 0.177 | 0.765 | 1.363 |
| P08103 | Hck | P15655 | Fgf2 | 48.1 | 34.9 | 103 | 94.8 | 104 | 0.42 | 0.175 | 0.765 | 1.36 |
| Q03146 | Ddr1 | P15655 | Fgf2 | 48 | 36.5 | 103 | 94.8 | 105 | 0.419 | 0.174 | 0.765 | 1.358 |
| P16056 | Met | P15655 | Fgf2 | 47.7 | 37.2 | 103 | 94.8 | 106 | 0.419 | 0.172 | 0.765 | 1.356 |
| P25911 | Lyn | P15655 | Fgf2 | 47.7 | 36.1 | 103 | 94.8 | 107 | 0.419 | 0.17 | 0.765 | 1.354 |
| Q62371 | Ddr2 | P15655 | Fgf2 | 47 | 36.5 | 103 | 94.8 | 108 | 0.417 | 0.169 | 0.765 | 1.351 |
| Q61772 | Epha7 | P15655 | Fgf2 | 47 | 34.1 | 103 | 94.8 | 109 | 0.417 | 0.167 | 0.765 | 1.349 |
| Q60750 | Epha1 | P15655 | Fgf2 | 47 | 33.6 | 103 | 94.8 | 110 | 0.417 | 0.165 | 0.765 | 1.347 |
| P05480 | Src | P15655 | Fgf2 | 46.7 | 36.1 | 103 | 94.8 | 111 | 0.416 | 0.164 | 0.765 | 1.345 |
| Q01887 | Ryk | P15655 | Fgf2 | 46.7 | 30.1 | 103 | 94.8 | 112 | 0.416 | 0.162 | 0.765 | 1.343 |
| P24604 | Tec | P15655 | Fgf2 | 46.2 | 34.7 | 103 | 94.8 | 113 | 0.415 | 0.161 | 0.765 | 1.341 |
| Q60629 | Epha5 | P15655 | Fgf2 | 46.1 | 34.9 | 103 | 94.8 | 114 | 0.414 | 0.159 | 0.765 | 1.338 |
| Q03145 | Epha2 | P15655 | Fgf2 | 46 | 35.6 | 103 | 94.8 | 115 | 0.414 | 0.157 | 0.765 | 1.336 |
| P29319 | Epha3 | P15655 | Fgf2 | 46 | 34.6 | 103 | 94.8 | 116 | 0.414 | 0.156 | 0.765 | 1.335 |
| P54763 | Ephb2 | P15655 | Fgf2 | 46 | 34.3 | 103 | 94.8 | 117 | 0.414 | 0.154 | 0.765 | 1.333 |
| P05622 | Pdgfrb | P15655 | Fgf2 | 45.7 | 51.3 | 103 | 94.8 | 118 | 0.413 | 0.153 | 0.765 | 1.331 |
| Q03137 | Epha4 | P15655 | Fgf2 | 45.7 | 35.2 | 103 | 94.8 | 119 | 0.413 | 0.151 | 0.765 | 1.329 |
| Q04736 | Yes1 | P15655 | Fgf2 | 45.5 | 36.4 | 103 | 94.8 | 120 | 0.413 | 0.15 | 0.765 | 1.328 |
| P16277 | Blk | P15655 | Fgf2 | 45.5 | 35.5 | 103 | 94.8 | 121 | 0.413 | 0.148 | 0.765 | 1.326 |
| Q9QVP9 | Ptk2b | P15655 | Fgf2 | 45.4 | 36.6 | 103 | 94.8 | 122 | 0.412 | 0.147 | 0.765 | 1.324 |
| Q8CBF3 | Ephb1 | P15655 | Fgf2 | 44.5 | 34 | 103 | 94.8 | 123 | 0.41 | 0.145 | 0.765 | 1.32 |
| P42682 | Txk | P15655 | Fgf2 | 44.4 | 34.7 | 103 | 94.8 | 124 | 0.409 | 0.144 | 0.765 | 1.318 |
| P70424 | Erbb2 | P15655 | Fgf2 | 44 | 36.3 | 103 | 94.8 | 125 | 0.408 | 0.143 | 0.765 | 1.316 |
| P35991 | Btk | P15655 | Fgf2 | 44 | 34.2 | 103 | 94.8 | 126 | 0.408 | 0.141 | 0.765 | 1.314 |
| P54761 | Ephb4 | P15655 | Fgf2 | 43.7 | 33.7 | 103 | 94.8 | 127 | 0.407 | 0.14 | 0.765 | 1.312 |
| P39688 | Fyn | P15655 | Fgf2 | 43.5 | 36.1 | 103 | 94.8 | 128 | 0.407 | 0.138 | 0.765 | 1.31 |
| Q03526 | Itk | P15655 | Fgf2 | 43.1 | 35.1 | 103 | 94.8 | 129 | 0.406 | 0.137 | 0.765 | 1.308 |
| O09127 | Epha8 | P15655 | Fgf2 | 42.7 | 34.8 | 103 | 94.8 | 130 | 0.405 | 0.136 | 0.765 | 1.306 |
| Q62120 | Jak2 | P15655 | Fgf2 | 42.3 | 35.2 | 103 | 94.8 | 131 | 0.404 | 0.134 | 0.765 | 1.303 |
| P54754 | Ephb3 | P15655 | Fgf2 | 42.2 | 33.3 | 103 | 94.8 | 132 | 0.403 | 0.133 | 0.765 | 1.301 |
| Q61527 | Erbb4 | P15655 | Fgf2 | 41.7 | 35.1 | 103 | 94.8 | 133 | 0.402 | 0.132 | 0.765 | 1.299 |
| P43404 | Zap70 | P15655 | Fgf2 | 41.3 | 33.2 | 103 | 94.8 | 134 | 0.401 | 0.13 | 0.765 | 1.296 |
| Q01279 | Egfr | P15655 | Fgf2 | 40.7 | 33.3 | 103 | 94.8 | 135 | 0.399 | 0.129 | 0.765 | 1.293 |
| P14234 | Fgr | P15655 | Fgf2 | 40.4 | 34.6 | 103 | 94.8 | 136 | 0.398 | 0.128 | 0.765 | 1.291 |
| Q6J9G1 | Styk1 | P15655 | Fgf2 | 40 | 29.9 | 103 | 94.8 | 137 | 0.397 | 0.126 | 0.765 | 1.288 |
| P35546 | Ret | P61148 | Fgf1 | 98.2 | 50 | 44.7 | 53.5 | 138 | 0.397 | 0.125 | 0.765 | 1.287 |
| P52332 | Jak1 | P15655 | Fgf2 | 39.7 | 34.7 | 103 | 94.8 | 139 | 0.396 | 0.124 | 0.765 | 1.285 |
| P35918 | Kdr | P61148 | Fgf1 | 97.5 | 35.2 | 44.7 | 53.5 | 140 | 0.395 | 0.122 | 0.765 | 1.282 |
| Q91V87 | Fgfrl1 | P15655 | Fgf2 | 39.1 | 31.7 | 103 | 94.8 | 141 | 0.395 | 0.121 | 0.765 | 1.281 |
| O54967 | Tnk2 | P15655 | Fgf2 | 39 | 36.2 | 103 | 94.8 | 142 | 0.395 | 0.12 | 0.765 | 1.28 |
| P48025 | Syk | P15655 | Fgf2 | 37.2 | 36.2 | 103 | 94.8 | 143 | 0.389 | 0.119 | 0.765 | 1.273 |
| Q61526 | Erbb3 | P15655 | Fgf2 | 37 | 32.3 | 103 | 94.8 | 144 | 0.389 | 0.117 | 0.765 | 1.271 |
| Q62137 | Jak3 | P15655 | Fgf2 | 35.7 | 32.6 | 103 | 94.8 | 145 | 0.385 | 0.116 | 0.765 | 1.266 |
| Q62413 | Epha6 | P15655 | Fgf2 | 35.7 | 28.6 | 103 | 94.8 | 146 | 0.385 | 0.115 | 0.765 | 1.265 |
| Q8BYG9 | Epha10 | P15655 | Fgf2 | 35.4 | 31.7 | 103 | 94.8 | 147 | 0.384 | 0.114 | 0.765 | 1.263 |
| Q9R117 | Tyk2 | P15655 | Fgf2 | 35.1 | 33.1 | 103 | 94.8 | 148 | 0.384 | 0.113 | 0.765 | 1.262 |
| Q5XJV6 | Lmtk3 | P15655 | Fgf2 | 31 | 27.6 | 103 | 94.8 | 149 | 0.372 | 0.111 | 0.765 | 1.248 |
| Q1HKZ5 | Map3k13 | P15655 | Fgf2 | 30.3 | 31.4 | 103 | 94.8 | 150 | 0.37 | 0.11 | 0.765 | 1.245 |
| Q60700 | Map3k12 | P15655 | Fgf2 | 30.3 | 31.3 | 103 | 94.8 | 151 | 0.37 | 0.109 | 0.765 | 1.244 |
| P35969 | Flt1 | P61148 | Fgf1 | 87.7 | 46.6 | 44.7 | 53.5 | 152 | 0.368 | 0.108 | 0.765 | 1.241 |
| Q99ML2 | Tnk1 | P15655 | Fgf2 | 29 | 31.1 | 103 | 94.8 | 153 | 0.367 | 0.107 | 0.765 | 1.239 |
| P35917 | Flt4 | P61148 | Fgf1 | 87.1 | 34.6 | 44.7 | 53.5 | 154 | 0.366 | 0.106 | 0.765 | 1.237 |
| Q3U1V8 | Map3k9 | P15655 | Fgf2 | 28.5 | 31.7 | 103 | 94.8 | 155 | 0.365 | 0.104 | 0.765 | 1.234 |
| Q9ESL4 | Mltk | P15655 | Fgf2 | 26.7 | 29 | 103 | 94.8 | 156 | 0.36 | 0.103 | 0.765 | 1.228 |
| Q66L42 | Map3k10 | P15655 | Fgf2 | 26 | 32.3 | 103 | 94.8 | 157 | 0.358 | 0.102 | 0.765 | 1.225 |
| Q80XI6 | Map3k11 | P15655 | Fgf2 | 25.7 | 31.2 | 103 | 94.8 | 158 | 0.357 | 0.101 | 0.765 | 1.223 |
| O08644 | Ephb6 | P15655 | Fgf2 | 25.3 | 28.8 | 103 | 94.8 | 159 | 0.356 | 0.1 | 0.765 | 1.221 |
| Q80YE4 | Aatk | P15655 | Fgf2 | 23.7 | 28.3 | 103 | 94.8 | 160 | 0.352 | 0.099 | 0.765 | 1.216 |
| Q8VDG6 | Mlk4 | P15655 | Fgf2 | 23.2 | 30.5 | 103 | 94.8 | 161 | 0.351 | 0.098 | 0.765 | 1.214 |
| P53668 | Limk1 | P15655 | Fgf2 | 22.1 | 27.6 | 103 | 94.8 | 162 | 0.347 | 0.097 | 0.765 | 1.209 |
| Q62073 | Map3k7 | P15655 | Fgf2 | 21 | 28.6 | 103 | 94.8 | 163 | 0.344 | 0.095 | 0.765 | 1.204 |
| Q8VCT9 | Tesk2 | P15655 | Fgf2 | 20.7 | 25.5 | 103 | 94.8 | 164 | 0.344 | 0.094 | 0.765 | 1.203 |
| Q99N57 | Raf1 | P15655 | Fgf2 | 20.5 | 27 | 103 | 94.8 | 165 | 0.343 | 0.093 | 0.765 | 1.201 |
| Q7TSC3 | Nek5 | P15655 | Fgf2 | 20.2 | 25.4 | 103 | 94.8 | 166 | 0.342 | 0.092 | 0.765 | 1.199 |
| P05532 | Kit | P61148 | Fgf1 | 78.5 | 42.4 | 44.7 | 53.5 | 167 | 0.342 | 0.091 | 0.765 | 1.198 |
| P28028 | Braf | P15655 | Fgf2 | 20.1 | 26.9 | 103 | 94.8 | 168 | 0.342 | 0.09 | 0.765 | 1.197 |
| P04627 | Araf | P15655 | Fgf2 | 18.7 | 26.9 | 103 | 94.8 | 169 | 0.338 | 0.089 | 0.765 | 1.192 |
| Q3TYD6 | Lmtk2 | P15655 | Fgf2 | 18.5 | 25.2 | 103 | 94.8 | 170 | 0.338 | 0.088 | 0.765 | 1.191 |
| Q02111 | Prkcq | P15655 | Fgf2 | 18 | 28.9 | 103 | 94.8 | 171 | 0.336 | 0.087 | 0.765 | 1.188 |
| P09581 | Csf1r | P61148 | Fgf1 | 76.3 | 42.5 | 44.7 | 53.5 | 172 | 0.336 | 0.086 | 0.765 | 1.187 |
| P18654 | Rps6ka3 | P15655 | Fgf2 | 17.7 | 29.5 | 103 | 94.8 | 173 | 0.335 | 0.085 | 0.765 | 1.185 |
| O70146 | Tesk1 | P15655 | Fgf2 | 17.5 | 24.8 | 103 | 94.8 | 174 | 0.335 | 0.084 | 0.765 | 1.184 |
| Q9ES74 | Nek7 | P15655 | Fgf2 | 17.4 | 25.4 | 103 | 94.8 | 175 | 0.334 | 0.083 | 0.765 | 1.182 |
| E9Q1V8 | Gm5174 | P15655 | Fgf2 | 17.2 | 29.6 | 103 | 94.8 | 176 | 0.334 | 0.082 | 0.765 | 1.181 |
| P35546 | Ret | P21658 | Fgf6 | 98.2 | 50 | 22 | 43.7 | 177 | 0.334 | 0.081 | 0.765 | 1.18 |
| O54785 | Limk2 | P15655 | Fgf2 | 16.7 | 27.8 | 103 | 94.8 | 178 | 0.332 | 0.08 | 0.765 | 1.177 |
| Q9ES70 | Nek6 | P15655 | Fgf2 | 16.7 | 27.1 | 103 | 94.8 | 179 | 0.332 | 0.079 | 0.765 | 1.176 |
| P35918 | Kdr | P21658 | Fgf6 | 97.5 | 35.2 | 22 | 43.7 | 180 | 0.332 | 0.078 | 0.765 | 1.175 |
| Q99KH8 | Stk24 | P15655 | Fgf2 | 16.4 | 27.9 | 103 | 94.8 | 181 | 0.332 | 0.077 | 0.765 | 1.174 |
| O70126 | Aurkb | P15655 | Fgf2 | 16.3 | 29.1 | 103 | 94.8 | 182 | 0.331 | 0.076 | 0.765 | 1.172 |
| Q9WUT3 | Rps6ka2 | P15655 | Fgf2 | 16.2 | 27.2 | 103 | 94.8 | 183 | 0.331 | 0.075 | 0.765 | 1.171 |
| Q9JI10 | Stk3 | P15655 | Fgf2 | 16.2 | 25.8 | 103 | 94.8 | 184 | 0.331 | 0.074 | 0.765 | 1.17 |
| Q9JI11 | Stk4 | P15655 | Fgf2 | 16 | 25.8 | 103 | 94.8 | 185 | 0.331 | 0.073 | 0.765 | 1.169 |
| Q4FZD7 | Plk5 | P15655 | Fgf2 | 16 | 25.2 | 103 | 94.8 | 186 | 0.331 | 0.072 | 0.765 | 1.168 |
| E9Q4Z5 | Gm6729 | P15655 | Fgf2 | 15.7 | 26.9 | 103 | 94.8 | 187 | 0.33 | 0.071 | 0.765 | 1.166 |
| Q61097 | Ksr1 | P15655 | Fgf2 | 15.7 | 23.2 | 103 | 94.8 | 188 | 0.33 | 0.07 | 0.765 | 1.165 |
| P35546 | Ret | P15656 | Fgf5 | 98.2 | 50 | 19.7 | 43 | 189 | 0.327 | 0.069 | 0.765 | 1.161 |
| P35546 | Ret | P05524 | Fgf3 | 98.2 | 50 | 19.7 | 41.6 | 190 | 0.327 | 0.068 | 0.765 | 1.16 |
| P35546 | Ret | P11403 | Fgf4 | 98.2 | 50 | 19.1 | 42 | 191 | 0.326 | 0.067 | 0.765 | 1.158 |
| P35918 | Kdr | P15656 | Fgf5 | 97.5 | 35.2 | 19.7 | 43 | 192 | 0.326 | 0.066 | 0.765 | 1.157 |
| P35918 | Kdr | P05524 | Fgf3 | 97.5 | 35.2 | 19.7 | 41.6 | 193 | 0.326 | 0.065 | 0.765 | 1.156 |
| P35918 | Kdr | P11403 | Fgf4 | 97.5 | 35.2 | 19.1 | 42 | 194 | 0.324 | 0.065 | 0.765 | 1.154 |
| P35546 | Ret | Q9ESL9 | Fgf20 | 98.2 | 50 | 16.5 | 37.5 | 195 | 0.319 | 0.064 | 0.765 | 1.148 |
| P35546 | Ret | P54130 | Fgf9 | 98.2 | 50 | 16.3 | 39.4 | 196 | 0.318 | 0.063 | 0.765 | 1.146 |
| P35918 | Kdr | Q9ESL9 | Fgf20 | 97.5 | 35.2 | 16.5 | 37.5 | 197 | 0.317 | 0.062 | 0.765 | 1.144 |
| Q02858 | Tek | P61148 | Fgf1 | 67.7 | 46.2 | 44.7 | 53.5 | 198 | 0.312 | 0.061 | 0.765 | 1.138 |
| Q06806 | Tie1 | P61148 | Fgf1 | 67.7 | 45.4 | 44.7 | 53.5 | 199 | 0.312 | 0.06 | 0.765 | 1.137 |
| P35969 | Flt1 | P21658 | Fgf6 | 87.7 | 46.6 | 22 | 43.7 | 200 | 0.305 | 0.059 | 0.765 | 1.129 |
| P35917 | Flt4 | P21658 | Fgf6 | 87.1 | 34.6 | 22 | 43.7 | 201 | 0.303 | 0.058 | 0.765 | 1.126 |
| P35969 | Flt1 | P15656 | Fgf5 | 87.7 | 46.6 | 19.7 | 43 | 202 | 0.298 | 0.057 | 0.765 | 1.12 |
| P35969 | Flt1 | P05524 | Fgf3 | 87.7 | 46.6 | 19.7 | 41.6 | 203 | 0.298 | 0.056 | 0.765 | 1.119 |
| P35969 | Flt1 | P11403 | Fgf4 | 87.7 | 46.6 | 19.1 | 42 | 204 | 0.297 | 0.056 | 0.765 | 1.118 |
| P35917 | Flt4 | P15656 | Fgf5 | 87.1 | 34.6 | 19.7 | 43 | 205 | 0.297 | 0.055 | 0.765 | 1.117 |
| P35917 | Flt4 | P05524 | Fgf3 | 87.1 | 34.6 | 19.7 | 41.6 | 206 | 0.297 | 0.054 | 0.765 | 1.116 |
| P35917 | Flt4 | P11403 | Fgf4 | 87.1 | 34.6 | 19.1 | 42 | 207 | 0.295 | 0.053 | 0.765 | 1.113 |
| Q60751 | Igf1r | P61148 | Fgf1 | 60.1 | 40.1 | 44.7 | 53.5 | 208 | 0.291 | 0.052 | 0.765 | 1.108 |
| Q6VNS1 | Ntrk3 | P61148 | Fgf1 | 59.7 | 27.1 | 44.7 | 53.5 | 209 | 0.29 | 0.051 | 0.765 | 1.106 |
| Q8BKG3 | Ptk7 | P61148 | Fgf1 | 58.7 | 27.8 | 44.7 | 53.5 | 210 | 0.287 | 0.05 | 0.765 | 1.102 |
| P00520 | Abl1 | P61148 | Fgf1 | 58.2 | 39.5 | 44.7 | 53.5 | 211 | 0.286 | 0.05 | 0.765 | 1.101 |
| Q4JIM5 | Abl2 | P61148 | Fgf1 | 58.1 | 39.9 | 44.7 | 53.5 | 212 | 0.286 | 0.049 | 0.765 | 1.1 |
| Q9WTL4 | Insrr | P61148 | Fgf1 | 57.7 | 37.8 | 44.7 | 53.5 | 213 | 0.284 | 0.048 | 0.765 | 1.097 |
| Q61006 | Musk | P61148 | Fgf1 | 56.5 | 40.2 | 44.7 | 53.5 | 214 | 0.281 | 0.047 | 0.765 | 1.093 |
| P15208 | Insr | P61148 | Fgf1 | 56.3 | 39.6 | 44.7 | 53.5 | 215 | 0.281 | 0.046 | 0.765 | 1.092 |
| Q62270 | Srms | P61148 | Fgf1 | 56.1 | 38.2 | 44.7 | 53.5 | 216 | 0.28 | 0.045 | 0.765 | 1.09 |
| P05532 | Kit | P21658 | Fgf6 | 78.5 | 42.4 | 22 | 43.7 | 217 | 0.279 | 0.045 | 0.765 | 1.089 |
| P70451 | Fer | P61148 | Fgf1 | 55.5 | 38.3 | 44.7 | 53.5 | 218 | 0.278 | 0.044 | 0.765 | 1.087 |
| P41242 | Matk | P61148 | Fgf1 | 55.2 | 39.2 | 44.7 | 53.5 | 219 | 0.278 | 0.043 | 0.765 | 1.086 |
| P15209 | Ntrk2 | P61148 | Fgf1 | 55.2 | 27.1 | 44.7 | 53.5 | 220 | 0.278 | 0.042 | 0.765 | 1.085 |
| Q3UFB7 | Ntrk1 | P61148 | Fgf1 | 54.7 | 36.9 | 44.7 | 53.5 | 221 | 0.276 | 0.041 | 0.765 | 1.082 |
| P09581 | Csf1r | P21658 | Fgf6 | 76.3 | 42.5 | 22 | 43.7 | 222 | 0.273 | 0.041 | 0.765 | 1.079 |
| P41241 | Csk | P61148 | Fgf1 | 53.5 | 37.2 | 44.7 | 53.5 | 223 | 0.273 | 0.04 | 0.765 | 1.078 |
| Q00993 | Axl | P61148 | Fgf1 | 53.4 | 37.9 | 44.7 | 53.5 | 224 | 0.272 | 0.039 | 0.765 | 1.076 |
| P97793 | Alk | P61148 | Fgf1 | 53.3 | 37 | 44.7 | 53.5 | 225 | 0.272 | 0.038 | 0.765 | 1.075 |
| Q64434 | Ptk6 | P61148 | Fgf1 | 53 | 42 | 44.7 | 53.5 | 226 | 0.271 | 0.037 | 0.765 | 1.073 |
| Q00342 | Flt3 | P61148 | Fgf1 | 53 | 36.4 | 44.7 | 53.5 | 227 | 0.271 | 0.037 | 0.765 | 1.073 |
| Q60805 | Mertk | P61148 | Fgf1 | 52.7 | 33.7 | 44.7 | 53.5 | 228 | 0.271 | 0.036 | 0.765 | 1.072 |
| Q922K9 | Frk | P61148 | Fgf1 | 52.4 | 40.3 | 44.7 | 53.5 | 229 | 0.27 | 0.035 | 0.765 | 1.07 |
| P55144 | Tyro3 | P61148 | Fgf1 | 52.4 | 37.2 | 44.7 | 53.5 | 230 | 0.27 | 0.034 | 0.765 | 1.069 |
| Q78DX7 | Ros1 | P61148 | Fgf1 | 52.3 | 40.9 | 44.7 | 53.5 | 231 | 0.269 | 0.034 | 0.765 | 1.068 |
| P08923 | Ltk | P61148 | Fgf1 | 52 | 36.8 | 44.7 | 53.5 | 232 | 0.269 | 0.033 | 0.765 | 1.067 |
| P16879 | Fes | P61148 | Fgf1 | 51.7 | 35.3 | 44.7 | 53.5 | 233 | 0.268 | 0.032 | 0.765 | 1.065 |
| Q9Z138 | Ror2 | P61148 | Fgf1 | 50.7 | 32.1 | 44.7 | 53.5 | 234 | 0.265 | 0.031 | 0.765 | 1.061 |
| P26618 | Pdgfra | P61148 | Fgf1 | 50.1 | 53.9 | 44.7 | 53.5 | 235 | 0.263 | 0.03 | 0.765 | 1.058 |
| P34152 | Ptk2 | P61148 | Fgf1 | 50 | 34.2 | 44.7 | 53.5 | 236 | 0.263 | 0.03 | 0.765 | 1.058 |
| Q9Z139 | Ror1 | P61148 | Fgf1 | 49.7 | 35.1 | 44.7 | 53.5 | 237 | 0.262 | 0.029 | 0.765 | 1.056 |
| P06240 | Lck | P61148 | Fgf1 | 49.2 | 38.3 | 44.7 | 53.5 | 238 | 0.261 | 0.028 | 0.765 | 1.054 |
| Q62190 | Mst1r | P61148 | Fgf1 | 49 | 34 | 44.7 | 53.5 | 239 | 0.26 | 0.027 | 0.765 | 1.052 |
| P97504 | Bmx | P61148 | Fgf1 | 48.5 | 35.7 | 44.7 | 53.5 | 240 | 0.259 | 0.027 | 0.765 | 1.051 |
| P08103 | Hck | P61148 | Fgf1 | 48.1 | 34.9 | 44.7 | 53.5 | 241 | 0.258 | 0.026 | 0.765 | 1.049 |
| Q03146 | Ddr1 | P61148 | Fgf1 | 48 | 36.5 | 44.7 | 53.5 | 242 | 0.257 | 0.025 | 0.765 | 1.047 |
| P16056 | Met | P61148 | Fgf1 | 47.7 | 37.2 | 44.7 | 53.5 | 243 | 0.257 | 0.025 | 0.765 | 1.047 |
| P25911 | Lyn | P61148 | Fgf1 | 47.7 | 36.1 | 44.7 | 53.5 | 244 | 0.257 | 0.024 | 0.765 | 1.046 |
| Q62371 | Ddr2 | P61148 | Fgf1 | 47 | 36.5 | 44.7 | 53.5 | 245 | 0.255 | 0.023 | 0.765 | 1.043 |
| Q61772 | Epha7 | P61148 | Fgf1 | 47 | 34.1 | 44.7 | 53.5 | 246 | 0.255 | 0.022 | 0.765 | 1.042 |
| Q60750 | Epha1 | P61148 | Fgf1 | 47 | 33.6 | 44.7 | 53.5 | 247 | 0.255 | 0.022 | 0.765 | 1.042 |
| P05480 | Src | P61148 | Fgf1 | 46.7 | 36.1 | 44.7 | 53.5 | 248 | 0.254 | 0.021 | 0.765 | 1.04 |
| Q01887 | Ryk | P61148 | Fgf1 | 46.7 | 30.1 | 44.7 | 53.5 | 249 | 0.254 | 0.02 | 0.765 | 1.039 |
| P24604 | Tec | P61148 | Fgf1 | 46.2 | 34.7 | 44.7 | 53.5 | 250 | 0.253 | 0.019 | 0.765 | 1.037 |
| Q60629 | Epha5 | P61148 | Fgf1 | 46.1 | 34.9 | 44.7 | 53.5 | 251 | 0.252 | 0.019 | 0.765 | 1.036 |
| Q03145 | Epha2 | P61148 | Fgf1 | 46 | 35.6 | 44.7 | 53.5 | 252 | 0.252 | 0.018 | 0.765 | 1.035 |
| P29319 | Epha3 | P61148 | Fgf1 | 46 | 34.6 | 44.7 | 53.5 | 253 | 0.252 | 0.017 | 0.765 | 1.034 |
| P54763 | Ephb2 | P61148 | Fgf1 | 46 | 34.3 | 44.7 | 53.5 | 254 | 0.252 | 0.017 | 0.765 | 1.034 |
| P05622 | Pdgfrb | P61148 | Fgf1 | 45.7 | 51.3 | 44.7 | 53.5 | 255 | 0.251 | 0.016 | 0.765 | 1.032 |
| Q03137 | Epha4 | P61148 | Fgf1 | 45.7 | 35.2 | 44.7 | 53.5 | 256 | 0.251 | 0.015 | 0.765 | 1.031 |
| Q04736 | Yes1 | P61148 | Fgf1 | 45.5 | 36.4 | 44.7 | 53.5 | 257 | 0.251 | 0.015 | 0.765 | 1.031 |
| P16277 | Blk | P61148 | Fgf1 | 45.5 | 35.5 | 44.7 | 53.5 | 258 | 0.251 | 0.014 | 0.765 | 1.03 |
| Q9QVP9 | Ptk2b | P61148 | Fgf1 | 45.4 | 36.6 | 44.7 | 53.5 | 259 | 0.25 | 0.013 | 0.765 | 1.028 |
| Q8CBF3 | Ephb1 | P61148 | Fgf1 | 44.5 | 34 | 44.7 | 53.5 | 260 | 0.248 | 0.013 | 0.765 | 1.026 |
| P42682 | Txk | P61148 | Fgf1 | 44.4 | 34.7 | 44.7 | 53.5 | 261 | 0.247 | 0.012 | 0.765 | 1.024 |
| P70424 | Erbb2 | P61148 | Fgf1 | 44 | 36.3 | 44.7 | 53.5 | 262 | 0.246 | 0.011 | 0.765 | 1.022 |
| P35991 | Btk | P61148 | Fgf1 | 44 | 34.2 | 44.7 | 53.5 | 263 | 0.246 | 0.01 | 0.765 | 1.021 |
| P54761 | Ephb4 | P61148 | Fgf1 | 43.7 | 33.7 | 44.7 | 53.5 | 264 | 0.246 | 0.01 | 0.765 | 1.021 |
| P39688 | Fyn | P61148 | Fgf1 | 43.5 | 36.1 | 44.7 | 53.5 | 265 | 0.245 | 0.009 | 0.765 | 1.019 |
| Q03526 | Itk | P61148 | Fgf1 | 43.1 | 35.1 | 44.7 | 53.5 | 266 | 0.244 | 0.008 | 0.765 | 1.017 |
| O09127 | Epha8 | P61148 | Fgf1 | 42.7 | 34.8 | 44.7 | 53.5 | 267 | 0.243 | 0.008 | 0.765 | 1.016 |
| Q62120 | Jak2 | P61148 | Fgf1 | 42.3 | 35.2 | 44.7 | 53.5 | 268 | 0.242 | 0.007 | 0.765 | 1.014 |
| P54754 | Ephb3 | P61148 | Fgf1 | 42.2 | 33.3 | 44.7 | 53.5 | 269 | 0.241 | 0.006 | 0.765 | 1.012 |
| Q61527 | Erbb4 | P61148 | Fgf1 | 41.7 | 35.1 | 44.7 | 53.5 | 270 | 0.24 | 0.006 | 0.765 | 1.011 |
| P43404 | Zap70 | P61148 | Fgf1 | 41.3 | 33.2 | 44.7 | 53.5 | 271 | 0.239 | 0.005 | 0.765 | 1.009 |
| Q01279 | Egfr | P61148 | Fgf1 | 40.7 | 33.3 | 44.7 | 53.5 | 272 | 0.237 | 0.005 | 0.765 | 1.007 |
| P14234 | Fgr | P61148 | Fgf1 | 40.4 | 34.6 | 44.7 | 53.5 | 273 | 0.236 | 0.004 | 0.765 | 1.005 |
| Q6J9G1 | Styk1 | P61148 | Fgf1 | 40 | 29.9 | 44.7 | 53.5 | 274 | 0.235 | 0.003 | 0.765 | 1.003 |
| P52332 | Jak1 | P61148 | Fgf1 | 39.7 | 34.7 | 44.7 | 53.5 | 275 | 0.234 | 0.003 | 0.765 | 1.002 |
| Q91V87 | Fgfrl1 | P61148 | Fgf1 | 39.1 | 31.7 | 44.7 | 53.5 | 276 | 0.233 | 0.002 | 0.765 | 1 |
| O54967 | Tnk2 | P61148 | Fgf1 | 39 | 36.2 | 44.7 | 53.5 | 277 | 0.233 | 0.001 | 0.765 | 0.999 |
| P48025 | Syk | P61148 | Fgf1 | 37.2 | 36.2 | 44.7 | 53.5 | 278 | 0.227 | 0.001 | 0.765 | 0.993 |
| Q61526 | Erbb3 | P61148 | Fgf1 | 37 | 32.3 | 44.7 | 53.5 | 279 | 0.227 | 0 | 0.765 | 0.992 |

^*^Sequence identity (SI)
