# Supplementary material for: Reconstructing Genome-Wide Protein–Protein Interaction Networks Using Multiple Strategies with Homologous Mapping
Source: PLoS One. 2015 Jan 20;10(1):e0116347. doi: 10.1371/journal.pone.0116347 (PMC4300222; doi:10.1371/journal.pone.0116347)
Supplement: S4 Table — (DOCX) [file pone.0116347.s004.docx]

**Table S4. The homologous PPIs derived from shot-p115**

| Protein  (A') | Gene name | Protein (B') | Gene name | *E*-value (A') | SI* (A') | *E*-value (B') | SI* (B') | Rank | *S_sim_* | *S_rank_* | *S_con_* | *S* |
| --- | --- | --- | --- | --- | --- | --- | --- | --- | --- | --- | --- | --- |
| Q9Z1Z0 | Uso1 | Q9QXS1 | Plec | 180 | 48.395 | 180 | 33.457 | 1 | 1 | 1 | 0.235 | 2.235 |
| Q9Z1Z0 | Uso1 | Q91ZU6 | Dst | 180 | 48.395 | 180 | 31.445 | 2 | 1 | 0.8 | 0.235 | 2.035 |
| Q9Z1Z0 | Uso1 | Q9QXZ0 | Macf1 | 180 | 48.395 | 180 | 31.099 | 3 | 1 | 0.683 | 0.235 | 1.918 |
| Q9Z1Z0 | Uso1 | Q62261 | Sptbn1 | 180 | 48.395 | 96.699 | 31.658 | 4 | 0.769 | 0.6 | 0.235 | 1.604 |
| Q9Z1Z0 | Uso1 | Q68FG2 | Sptbn2 | 180 | 48.395 | 92 | 32.789 | 5 | 0.756 | 0.536 | 0.235 | 1.527 |
| Q9Z1Z0 | Uso1 | P15508 | Sptb | 180 | 48.395 | 88 | 26.9 | 6 | 0.744 | 0.483 | 0.235 | 1.462 |
| Q9Z1Z0 | Uso1 | O88990 | Actn3 | 180 | 48.395 | 73.699 | 37.393 | 7 | 0.705 | 0.439 | 0.235 | 1.379 |
| Q9Z1Z0 | Uso1 | E9PX29 | Sptbn4 | 180 | 48.395 | 73.222 | 30.845 | 8 | 0.703 | 0.4 | 0.235 | 1.338 |
| Q9Z1Z0 | Uso1 | Q91ZE6 | Sptbn4 | 180 | 48.395 | 73 | 34.926 | 9 | 0.703 | 0.366 | 0.235 | 1.304 |
| Q9Z1Z0 | Uso1 | P57780 | Actn4 | 180 | 48.395 | 70.523 | 36.596 | 10 | 0.696 | 0.336 | 0.235 | 1.267 |
| Q9Z1Z0 | Uso1 | Q9JI91 | Actn2 | 180 | 48.395 | 69 | 35.542 | 11 | 0.692 | 0.308 | 0.235 | 1.235 |
| Q9Z1Z0 | Uso1 | Q7TPR4 | Actn1 | 180 | 48.395 | 66.699 | 35.991 | 12 | 0.685 | 0.283 | 0.235 | 1.203 |
| Q9Z1Z0 | Uso1 | F7BYG3 | Sptbn5 | 180 | 48.395 | 65 | 28.09 | 13 | 0.681 | 0.26 | 0.235 | 1.176 |
| Q9Z1Z0 | Uso1 | P11531 | Dmd | 180 | 48.395 | 59.097 | 48.548 | 14 | 0.664 | 0.239 | 0.235 | 1.138 |
| Q9Z1Z0 | Uso1 | E9Q6R7 | Utrn | 180 | 48.395 | 57.699 | 45.122 | 15 | 0.66 | 0.219 | 0.235 | 1.114 |
| Q9Z1Z0 | Uso1 | Q6ZWR6 | Syne1 | 180 | 48.395 | 55 | 42.642 | 16 | 0.653 | 0.2 | 0.235 | 1.088 |
| Q9Z1Z0 | Uso1 | Q6ZWQ0 | Syne2 | 180 | 48.395 | 50 | 42.424 | 17 | 0.639 | 0.183 | 0.235 | 1.057 |
| Q9Z1Z0 | Uso1 | Q9R269 | Ppl | 180 | 48.395 | 46.523 | 35.652 | 18 | 0.629 | 0.166 | 0.235 | 1.03 |
| Q9Z1Z0 | Uso1 | Q8C5W0 | Clmn | 180 | 48.395 | 44.222 | 39.273 | 19 | 0.623 | 0.15 | 0.235 | 1.008 |
| Q9Z1Z0 | Uso1 | E9Q557 | Dsp | 180 | 48.395 | 38.398 | 27.273 | 20 | 0.607 | 0.136 | 0.235 | 0.978 |
| Q9Z1Z0 | Uso1 | Q8VHX6 | Flnc | 180 | 48.395 | 30.155 | 38.839 | 21 | 0.584 | 0.122 | 0.235 | 0.941 |
| Q9Z1Z0 | Uso1 | Q80X90 | Flnb | 180 | 48.395 | 30 | 37.946 | 22 | 0.583 | 0.108 | 0.235 | 0.926 |
| Q9Z1Z0 | Uso1 | Q8BTM8 | Flna | 180 | 48.395 | 27.699 | 36.607 | 23 | 0.577 | 0.095 | 0.235 | 0.907 |
| Q9Z1Z0 | Uso1 | Q9D952 | Evpl | 180 | 48.395 | 24.398 | 26.68 | 24 | 0.568 | 0.083 | 0.235 | 0.886 |
| Q9Z1Z0 | Uso1 | P16546 | Sptan1 | 180 | 48.395 | 17.699 | 20.011 | 25 | 0.549 | 0.071 | 0.235 | 0.855 |
| Q9Z1Z0 | Uso1 | Q8BGT6 | Micall1 | 180 | 48.395 | 17 | 46.226 | 26 | 0.547 | 0.06 | 0.235 | 0.842 |
| Q9Z1Z0 | Uso1 | Q8JZP9 | Gas2l1 | 180 | 48.395 | 14.301 | 39.024 | 27 | 0.54 | 0.049 | 0.235 | 0.824 |
| Q9Z1Z0 | Uso1 | Q3TN34 | Micall2 | 180 | 48.395 | 13.222 | 39.623 | 28 | 0.537 | 0.039 | 0.235 | 0.811 |
| Q9Z1Z0 | Uso1 | Q5SSG4 | Gas2l2 | 180 | 48.395 | 13.155 | 41.905 | 29 | 0.537 | 0.028 | 0.235 | 0.8 |
| Q9Z1Z0 | Uso1 | Q8CJ19 | Mical3 | 180 | 48.395 | 13.097 | 32.031 | 30 | 0.536 | 0.019 | 0.235 | 0.79 |
| Q9Z1Z0 | Uso1 | Q8BML1 | Mical2 | 180 | 48.395 | 11.222 | 35.577 | 31 | 0.531 | 0.009 | 0.235 | 0.775 |
| Q9Z1Z0 | Uso1 | D3YWH8 | Sptbn4 | 180 | 48.395 | 10.699 | 64.151 | 32 | 0.53 | 0 | 0.235 | 0.765 |

^*^Sequence identity (SI)
