# Supplementary material for: Reconstructing Genome-Wide Protein–Protein Interaction Networks Using Multiple Strategies with Homologous Mapping
Source: PLoS One. 2015 Jan 20;10(1):e0116347. doi: 10.1371/journal.pone.0116347 (PMC4300222; doi:10.1371/journal.pone.0116347)
Supplement: S5 Table — (DOCX) [file pone.0116347.s005.docx]

**Table S5. 216 pathways and 76 structural complexes derived from KEGG**

| KEGG ID | Type | Name |
| --- | --- | --- |
| mmu00010 | pathway | Glycolysis / Gluconeogenesis |
| mmu00020 | pathway | Citrate cycle (TCA cycle) |
| mmu00030 | pathway | Pentose phosphate pathway |
| mmu00040 | pathway | Pentose and glucuronate interconversions |
| mmu00051 | pathway | Fructose and mannose metabolism |
| mmu00052 | pathway | Galactose metabolism |
| mmu00053 | pathway | Ascorbate and aldarate metabolism |
| mmu00061 | pathway | Fatty acid biosynthesis |
| mmu00062 | pathway | Fatty acid elongation in mitochondria |
| mmu00071 | pathway | Fatty acid metabolism |
| mmu00072 | pathway | Synthesis and degradation of ketone bodies |
| mmu00100 | pathway | Steroid biosynthesis |
| mmu00120 | pathway | Primary bile acid biosynthesis |
| mmu00130 | pathway | Ubiquinone and other terpenoid-quinone biosynthesis |
| mmu00140 | pathway | Steroid hormone biosynthesis |
| mmu00190 | pathway | Oxidative phosphorylation |
| mmu00230 | pathway | Purine metabolism |
| mmu00232 | pathway | Caffeine metabolism |
| mmu00240 | pathway | Pyrimidine metabolism |
| mmu00250 | pathway | Alanine, aspartate and glutamate metabolism |
| mmu00260 | pathway | Glycine, serine and threonine metabolism |
| mmu00270 | pathway | Cysteine and methionine metabolism |
| mmu00280 | pathway | Valine, leucine and isoleucine degradation |
| mmu00290 | pathway | Valine, leucine and isoleucine biosynthesis |
| mmu00300 | pathway | Lysine biosynthesis |
| mmu00310 | pathway | Lysine degradation |
| mmu00330 | pathway | Arginine and proline metabolism |
| mmu00340 | pathway | Histidine metabolism |
| mmu00350 | pathway | Tyrosine metabolism |
| mmu00360 | pathway | Phenylalanine metabolism |
| mmu00380 | pathway | Tryptophan metabolism |
| mmu00400 | pathway | Phenylalanine, tyrosine and tryptophan biosynthesis |
| mmu00410 | pathway | beta-Alanine metabolism |
| mmu00430 | pathway | Taurine and hypotaurine metabolism |
| mmu00450 | pathway | Selenoamino acid metabolism |
| mmu00460 | pathway | Cyanoamino acid metabolism |
| mmu00471 | pathway | D-Glutamine and D-glutamate metabolism |
| mmu00472 | pathway | D-Arginine and D-ornithine metabolism |
| mmu00480 | pathway | Glutathione metabolism |
| mmu00500 | pathway | Starch and sucrose metabolism |
| mmu00510 | pathway | N-Glycan biosynthesis |
| mmu00511 | pathway | Other glycan degradation |
| mmu00512 | pathway | O-Glycan biosynthesis |
| mmu00514 | pathway | O-Mannosyl glycan biosynthesis |
| mmu00520 | pathway | Amino sugar and nucleotide sugar metabolism |
| mmu00524 | pathway | Butirosin and neomycin biosynthesis |
| mmu00531 | pathway | Glycosaminoglycan degradation |
| mmu00532 | pathway | Glycosaminoglycan biosynthesis |
| mmu00533 | pathway | Glycosaminoglycan biosynthesis |
| mmu00534 | pathway | Glycosaminoglycan biosynthesis |
| mmu00561 | pathway | Glycerolipid metabolism |
| mmu00562 | pathway | Inositol phosphate metabolism |
| mmu00563 | pathway | Glycosylphosphatidylinositol(GPI)-anchor biosynthesis |
| mmu00564 | pathway | Glycerophospholipid metabolism |
| mmu00565 | pathway | Ether lipid metabolism |
| mmu00590 | pathway | Arachidonic acid metabolism |
| mmu00591 | pathway | Linoleic acid metabolism |
| mmu00592 | pathway | alpha-Linolenic acid metabolism |
| mmu00600 | pathway | Sphingolipid metabolism |
| mmu00601 | pathway | Glycosphingolipid biosynthesis |
| mmu00603 | pathway | Glycosphingolipid biosynthesis |
| mmu00604 | pathway | Glycosphingolipid biosynthesis |
| mmu00620 | pathway | Pyruvate metabolism |
| mmu00630 | pathway | Glyoxylate and dicarboxylate metabolism |
| mmu00640 | pathway | Propanoate metabolism |
| mmu00650 | pathway | Butanoate metabolism |
| mmu00670 | pathway | One carbon pool by folate |
| mmu00730 | pathway | Thiamine metabolism |
| mmu00740 | pathway | Riboflavin metabolism |
| mmu00750 | pathway | Vitamin B6 metabolism |
| mmu00760 | pathway | Nicotinate and nicotinamide metabolism |
| mmu00770 | pathway | Pantothenate and CoA biosynthesis |
| mmu00780 | pathway | Biotin metabolism |
| mmu00785 | pathway | Lipoic acid metabolism |
| mmu00790 | pathway | Folate biosynthesis |
| mmu00830 | pathway | Retinol metabolism |
| mmu00860 | pathway | Porphyrin and chlorophyll metabolism |
| mmu00900 | pathway | Terpenoid backbone biosynthesis |
| mmu00910 | pathway | Nitrogen metabolism |
| mmu00920 | pathway | Sulfur metabolism |
| mmu00970 | pathway | Aminoacyl-tRNA biosynthesis |
| mmu00980 | pathway | Metabolism of xenobiotics by cytochrome P450 |
| mmu00982 | pathway | Drug metabolism |
| mmu00983 | pathway | Drug metabolism |
| mmu01040 | pathway | Biosynthesis of unsaturated fatty acids |
| mmu01100 | pathway | Metabolic pathways |
| mmu02010 | pathway | ABC transporters |
| mmu03010 | pathway | Ribosome |
| mmu03018 | pathway | RNA degradation |
| mmu03020 | pathway | RNA polymerase |
| mmu03022 | pathway | Basal transcription factors |
| mmu03030 | pathway | DNA replication |
| mmu03040 | pathway | Spliceosome |
| mmu03050 | pathway | Proteasome |
| mmu03060 | pathway | Protein export |
| mmu03320 | pathway | PPAR signaling pathway |
| mmu03410 | pathway | Base excision repair |
| mmu03420 | pathway | Nucleotide excision repair |
| mmu03430 | pathway | Mismatch repair |
| mmu03440 | pathway | Homologous recombination |
| mmu03450 | pathway | Non-homologous end-joining |
| mmu04010 | pathway | MAPK signaling pathway |
| mmu04012 | pathway | ErbB signaling pathway |
| mmu04020 | pathway | Calcium signaling pathway |
| mmu04060 | pathway | Cytokine-cytokine receptor interaction |
| mmu04062 | pathway | Chemokine signaling pathway |
| mmu04070 | pathway | Phosphatidylinositol signaling system |
| mmu04080 | pathway | Neuroactive ligand-receptor interaction |
| mmu04110 | pathway | Cell cycle |
| mmu04114 | pathway | Oocyte meiosis |
| mmu04115 | pathway | p53 signaling pathway |
| mmu04120 | pathway | Ubiquitin mediated proteolysis |
| mmu04122 | pathway | Sulfur relay system |
| mmu04130 | pathway | SNARE interactions in vesicular transport |
| mmu04140 | pathway | Regulation of autophagy |
| mmu04141 | pathway | Protein processing in endoplasmic reticulum |
| mmu04142 | pathway | Lysosome |
| mmu04144 | pathway | Endocytosis |
| mmu04145 | pathway | Phagosome |
| mmu04146 | pathway | Peroxisome |
| mmu04150 | pathway | mTOR signaling pathway |
| mmu04210 | pathway | Apoptosis |
| mmu04260 | pathway | Cardiac muscle contraction |
| mmu04270 | pathway | Vascular smooth muscle contraction |
| mmu04310 | pathway | Wnt signaling pathway |
| mmu04320 | pathway | Dorso-ventral axis formation |
| mmu04330 | pathway | Notch signaling pathway |
| mmu04340 | pathway | Hedgehog signaling pathway |
| mmu04350 | pathway | TGF-beta signaling pathway |
| mmu04360 | pathway | Axon guidance |
| mmu04370 | pathway | VEGF signaling pathway |
| mmu04510 | pathway | Focal adhesion |
| mmu04512 | pathway | ECM-receptor interaction |
| mmu04514 | pathway | Cell adhesion molecules (CAMs) |
| mmu04520 | pathway | Adherens junction |
| mmu04530 | pathway | Tight junction |
| mmu04540 | pathway | Gap junction |
| mmu04610 | pathway | Complement and coagulation cascades |
| mmu04612 | pathway | Antigen processing and presentation |
| mmu04614 | pathway | Renin-angiotensin system |
| mmu04620 | pathway | Toll-like receptor signaling pathway |
| mmu04621 | pathway | NOD-like receptor signaling pathway |
| mmu04622 | pathway | RIG-I-like receptor signaling pathway |
| mmu04623 | pathway | Cytosolic DNA-sensing pathway |
| mmu04630 | pathway | Jak-STAT signaling pathway |
| mmu04640 | pathway | Hematopoietic cell lineage |
| mmu04650 | pathway | Natural killer cell mediated cytotoxicity |
| mmu04660 | pathway | T cell receptor signaling pathway |
| mmu04662 | pathway | B cell receptor signaling pathway |
| mmu04664 | pathway | Fc epsilon RI signaling pathway |
| mmu04666 | pathway | Fc gamma R-mediated phagocytosis |
| mmu04670 | pathway | Leukocyte transendothelial migration |
| mmu04672 | pathway | Intestinal immune network for IgA production |
| mmu04710 | pathway | Circadian rhythm |
| mmu04720 | pathway | Long-term potentiation |
| mmu04722 | pathway | Neurotrophin signaling pathway |
| mmu04730 | pathway | Long-term depression |
| mmu04740 | pathway | Olfactory transduction |
| mmu04742 | pathway | Taste transduction |
| mmu04744 | pathway | Phototransduction |
| mmu04810 | pathway | Regulation of actin cytoskeleton |
| mmu04910 | pathway | Insulin signaling pathway |
| mmu04912 | pathway | GnRH signaling pathway |
| mmu04914 | pathway | Progesterone-mediated oocyte maturation |
| mmu04916 | pathway | Melanogenesis |
| mmu04920 | pathway | Adipocytokine signaling pathway |
| mmu04930 | pathway | Type II diabetes mellitus |
| mmu04940 | pathway | Type I diabetes mellitus |
| mmu04950 | pathway | Maturity onset diabetes of the young |
| mmu04960 | pathway | Aldosterone-regulated sodium reabsorption |
| mmu04962 | pathway | Vasopressin-regulated water reabsorption |
| mmu04964 | pathway | Proximal tubule bicarbonate reclamation |
| mmu04966 | pathway | Collecting duct acid secretion |
| mmu04970 | pathway | Salivary secretion |
| mmu04971 | pathway | Gastric acid secretion |
| mmu04972 | pathway | Pancreatic secretion |
| mmu04973 | pathway | Carbohydrate digestion and absorption |
| mmu04974 | pathway | Protein digestion and absorption |
| mmu05010 | pathway | Alzheimer's disease |
| mmu05012 | pathway | Parkinson's disease |
| mmu05014 | pathway | Amyotrophic lateral sclerosis (ALS) |
| mmu05016 | pathway | Huntington's disease |
| mmu05020 | pathway | Prion diseases |
| mmu05100 | pathway | Bacterial invasion of epithelial cells |
| mmu05140 | pathway | Leishmaniasis |
| mmu05142 | pathway | Chagas disease |
| mmu05144 | pathway | Malaria |
| mmu05145 | pathway | Toxoplasmosis |
| mmu05146 | pathway | Amoebiasis |
| mmu05150 | pathway | Staphylococcus aureus infection |
| mmu05160 | pathway | Hepatitis C |
| mmu05200 | pathway | Pathways in cancer |
| mmu05210 | pathway | Colorectal cancer |
| mmu05211 | pathway | Renal cell carcinoma |
| mmu05212 | pathway | Pancreatic cancer |
| mmu05213 | pathway | Endometrial cancer |
| mmu05214 | pathway | Glioma |
| mmu05215 | pathway | Prostate cancer |
| mmu05216 | pathway | Thyroid cancer |
| mmu05217 | pathway | Basal cell carcinoma |
| mmu05218 | pathway | Melanoma |
| mmu05219 | pathway | Bladder cancer |
| mmu05220 | pathway | Chronic myeloid leukemia |
| mmu05221 | pathway | Acute myeloid leukemia |
| mmu05222 | pathway | Small cell lung cancer |
| mmu05223 | pathway | Non-small cell lung cancer |
| mmu05310 | pathway | Asthma |
| mmu05320 | pathway | Autoimmune thyroid disease |
| mmu05322 | pathway | Systemic lupus erythematosus |
| mmu05330 | pathway | Allograft rejection |
| mmu05332 | pathway | Graft-versus-host disease |
| mmu05340 | pathway | Primary immunodeficiency |
| mmu05410 | pathway | Hypertrophic cardiomyopathy (HCM) |
| mmu05412 | pathway | Arrhythmogenic right ventricular cardiomyopathy (ARVC) |
| mmu05414 | pathway | Dilated cardiomyopathy |
| mmu05416 | pathway | Viral myocarditis |
| mmu_M00072 | Structural complex | Oligosaccharyltransferase |
| mmu_M00142 | Structural complex | NADH:ubiquinone oxidoreductase, mitochondria |
| mmu_M00143 | Structural complex | NADH dehydrogenase (ubiquinone) Fe-S protein/flavoprotein complex, mitochondria |
| mmu_M00146 | Structural complex | NADH dehydrogenase (ubiquinone) 1 alpha subcomplex |
| mmu_M00147 | Structural complex | NADH dehydrogenase (ubiquinone) 1 beta subcomplex |
| mmu_M00148 | Structural complex | Succinate dehydrogenase (ubiquinone) |
| mmu_M00151 | Structural complex | Cytochrome bc1 complex respiratory unit |
| mmu_M00152 | Structural complex | Cytochrome bc1 complex |
| mmu_M00154 | Structural complex | Cytochrome c oxidase |
| mmu_M00158 | Structural complex | F-type ATPase, eukaryotes |
| mmu_M00160 | Structural complex | V-type ATPase, eukaryotes |
| mmu_M00177 | Structural complex | Ribosome, eukaryotes |
| mmu_M00180 | Structural complex | RNA polymerase II, eukaryotes |
| mmu_M00181 | Structural complex | RNA polymerase III, eukaryotes |
| mmu_M00182 | Structural complex | RNA polymerase I, eukaryotes |
| mmu_M00261 | Structural complex | DNA polymerase alpha / primase complex |
| mmu_M00262 | Structural complex | DNA polymerase delta complex |
| mmu_M00263 | Structural complex | DNA polymerase epsilon complex |
| mmu_M00284 | Structural complex | Origin recognition complex |
| mmu_M00285 | Structural complex | MCM complex |
| mmu_M00286 | Structural complex | GINS complex |
| mmu_M00288 | Structural complex | RPA complex |
| mmu_M00289 | Structural complex | RF-C complex |
| mmu_M00290 | Structural complex | Holo-TFIIH complex |
| mmu_M00291 | Structural complex | MRN complex |
| mmu_M00293 | Structural complex | DNA polymerase zeta complex |
| mmu_M00294 | Structural complex | DNA polymerase gamma complex |
| mmu_M00295 | Structural complex | BRCA1-associated genome surveillance complex (BASC) |
| mmu_M00296 | Structural complex | BER complex |
| mmu_M00297 | Structural complex | DNA-PK complex |
| mmu_M00337 | Structural complex | Immunoproteasome |
| mmu_M00340 | Structural complex | Proteasome, 20S core particle |
| mmu_M00341 | Structural complex | Proteasome, 19S regulatory particle (PA700) |
| mmu_M00351 | Structural complex | Spliceosome, U1-snRNP |
| mmu_M00352 | Structural complex | Spliceosome, U2-snRNP |
| mmu_M00353 | Structural complex | Spliceosome, Prp19/CDC5L complex |
| mmu_M00354 | Structural complex | Spliceosome, U4/U6.U5 tri-snRNP |
| mmu_M00355 | Structural complex | Spliceosome, 35S U5-snRNP |
| mmu_M00380 | Structural complex | SCF-BTRC complex |
| mmu_M00381 | Structural complex | SCF-SKP2 complex |
| mmu_M00382 | Structural complex | SCF-FBS complex |
| mmu_M00383 | Structural complex | ECV complex |
| mmu_M00384 | Structural complex | Cul3-SPOP complex |
| mmu_M00385 | Structural complex | Cul4-DDB1-DDB2 complex |
| mmu_M00386 | Structural complex | Cul4-DDB1-CSA complex |
| mmu_M00387 | Structural complex | SCF-FBW7 complex |
| mmu_M00388 | Structural complex | ECS complex |
| mmu_M00389 | Structural complex | APC/C complex |
| mmu_M00390 | Structural complex | Exosome, archaea |
| mmu_M00391 | Structural complex | Exosome, eukaryotes |
| mmu_M00392 | Structural complex | Ski complex |
| mmu_M00393 | Structural complex | TRAMP complex |
| mmu_M00395 | Structural complex | Decapping complex |
| mmu_M00396 | Structural complex | Lsm 2-8 complex |
| mmu_M00397 | Structural complex | Lsm 1-7 complex |
| mmu_M00398 | Structural complex | Sm core complex |
| mmu_M00399 | Structural complex | Cap binding complex |
| mmu_M00400 | Structural complex | p97-Ufd1-Npl4 complex |
| mmu_M00401 | Structural complex | Sec61 complex |
| mmu_M00402 | Structural complex | Translocon-associated protein (TRAP) complex |
| mmu_M00403 | Structural complex | HRD1/SEL1 ERAD complex |
| mmu_M00404 | Structural complex | COPII complex |
| mmu_M00405 | Structural complex | THC complex |
| mmu_M00406 | Structural complex | TREX complex |
| mmu_M00408 | Structural complex | ESCRT-0 complex |
| mmu_M00409 | Structural complex | ESCRT-I complex |
| mmu_M00410 | Structural complex | ESCRT-II complex |
| mmu_M00412 | Structural complex | ESCRT-III complex |
| mmu_M00413 | Structural complex | FA core complex |
| mmu_M00414 | Structural complex | Bloom's syndrome complex |
| mmu_M00424 | Structural complex | Shelterin complex |
| mmu_M00425 | Structural complex | H/ACA ribonucleoprotein complex |
| mmu_M00426 | Structural complex | Survival motor neuron (SMN) complex |
| mmu_M00427 | Structural complex | Nuclear pore complex |
| mmu_M00428 | Structural complex | eIF4F complex |
| mmu_M00430 | Structural complex | Exon junction complex (EJC) |
