# Supplementary material for: Reconstructing Genome-Wide Protein–Protein Interaction Networks Using Multiple Strategies with Homologous Mapping
Source: PLoS One. 2015 Jan 20;10(1):e0116347. doi: 10.1371/journal.pone.0116347 (PMC4300222; doi:10.1371/journal.pone.0116347)
Supplement: S6 Table — (DOCX) [file pone.0116347.s006.docx]

**Table S6. 14 PPIs derived from our method in the Sm complex**

| Protein (A') | Gene name | Protein (B') | Gene name | *S_sim_* | *S_rank_* | *S_con_* | *S* |
| --- | --- | --- | --- | --- | --- | --- | --- |
| P62317 | Snrpd2 | P27048 | Snrpb | 0.914 | 1 | 0.765 | 2.679 |
| P62320 | Snrpd3 | P27048 | Snrpb | 0.917 | 1 | 0.765 | 2.682 |
| P62317 | Snrpd2 | P62307 | Snrpf | 0.738 | 1 | 1 | 2.738 |
| P62307 | Snrpf | P27048 | Snrpb | 0.896 | 1 | 0.765 | 2.661 |
| P62317 | Snrpd2 | P62315 | Snrpd1 | 0.912 | 1 | 0.765 | 2.677 |
| P62309 | Snrpg | P27048 | Snrpb | 0.888 | 1 | 0.765 | 2.653 |
| P62315 | Snrpd1 | P27048 | Snrpb | 0.913 | 1 | 0.765 | 2.678 |
| P62317 | Snrpd2 | P62305 | Snrpe | 0.901 | 1 | 0.765 | 2.666 |
| P62320 | Snrpd3 | P62305 | Snrpe | 0.904 | 1 | 0.765 | 2.669 |
| P62315 | Snrpd1 | P62305 | Snrpe | 0.9 | 1 | 0.765 | 2.665 |
| P62309 | Snrpg | P62305 | Snrpe | 0.874 | 1 | 0.765 | 2.639 |
| P62307 | Snrpf | P62305 | Snrpe | 0.883 | 1 | 1 | 2.883 |
| P62305 | Snrpe | P27048 | Snrpb | 0.747 | 1 | 1 | 2.747 |
| P62320 | Snrpd3 | P62309 | Snrpg | 0.89 | 1 | 0.765 | 2.655 |
