# Supplementary figures and images for: Reconstructing Genome-Wide Protein–Protein Interaction Networks Using Multiple Strategies with Homologous Mapping
Source: PLoS One. 2015 Jan 20;10(1):e0116347. doi: 10.1371/journal.pone.0116347 (PMC4300222; doi:10.1371/journal.pone.0116347)

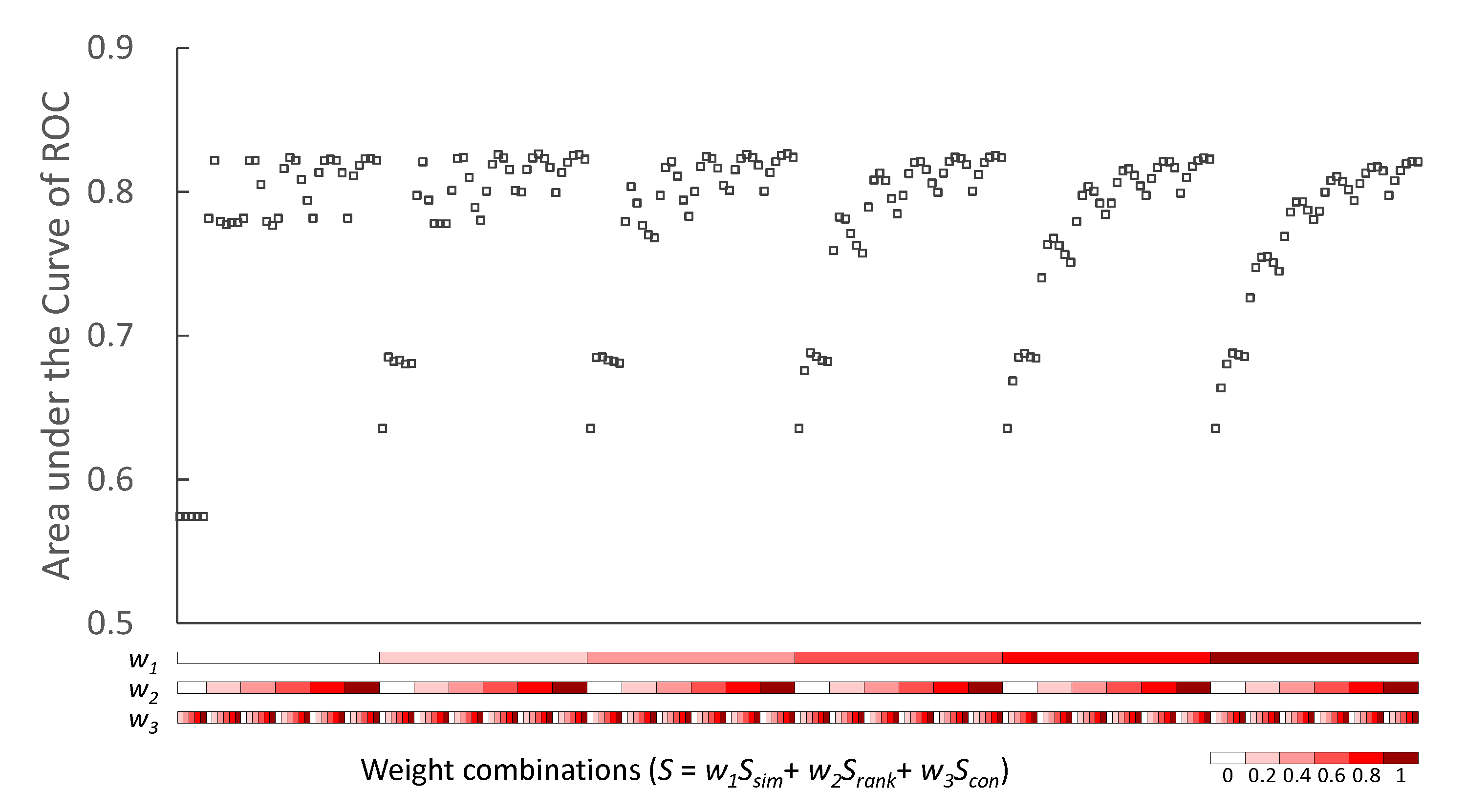

Supplement: S1 Fig — The w1, w2, and w3 values are tested by various values ranging from 0 to 1. Finally, the w1, w2, and w3 are set to 1. (TIF) [file pone.0116347.s007.tif]

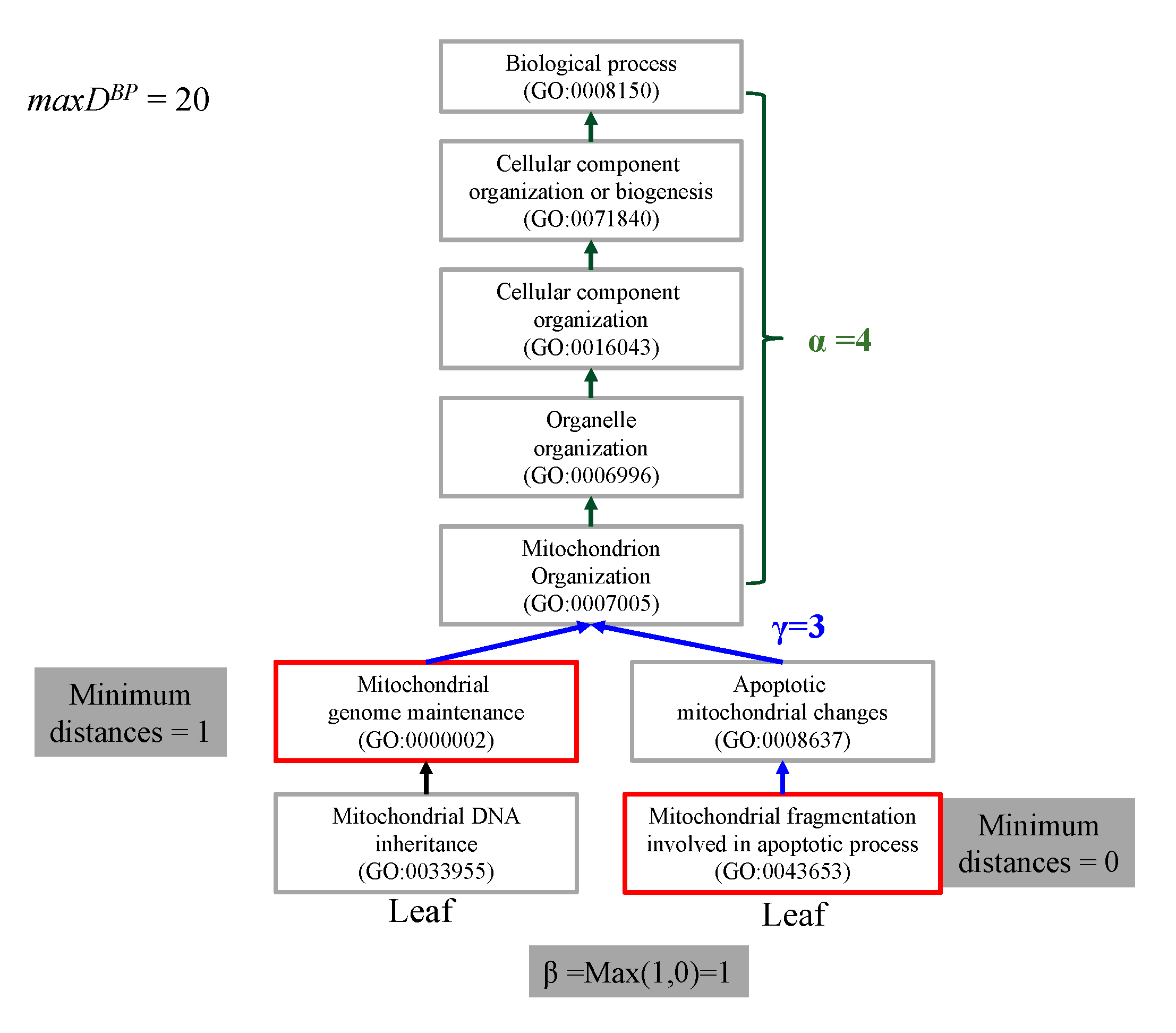

Supplement: S2 Fig — The RSS of two GO terms, ti and tj, is calculated by: RSS(ti,tj)=maxDGOmaxDGO+γ×αα+β, where maxD GO is the maximum depth from the root term of the GO to the leaf terms (i.e., maxDBP = 20 and maxDCC = 18 based on data version: releases 2014-05-29); α is the depth from the root term to most recent common ancestor (MRCA) of ti and tj; β is the max(DLi, DLj), where DLi and DLj are the minimum depths from ti to its leaf terms and tj to its leaf terms, respectively; γ is sum of distances between MRCA to ti and MRCA to tj. Therefore, the RSS (GO:0000002, GO:0043653) is (20/(20+3)) × (4/(4+1)) = 0.696. (TIF) [file pone.0116347.s008.tif]

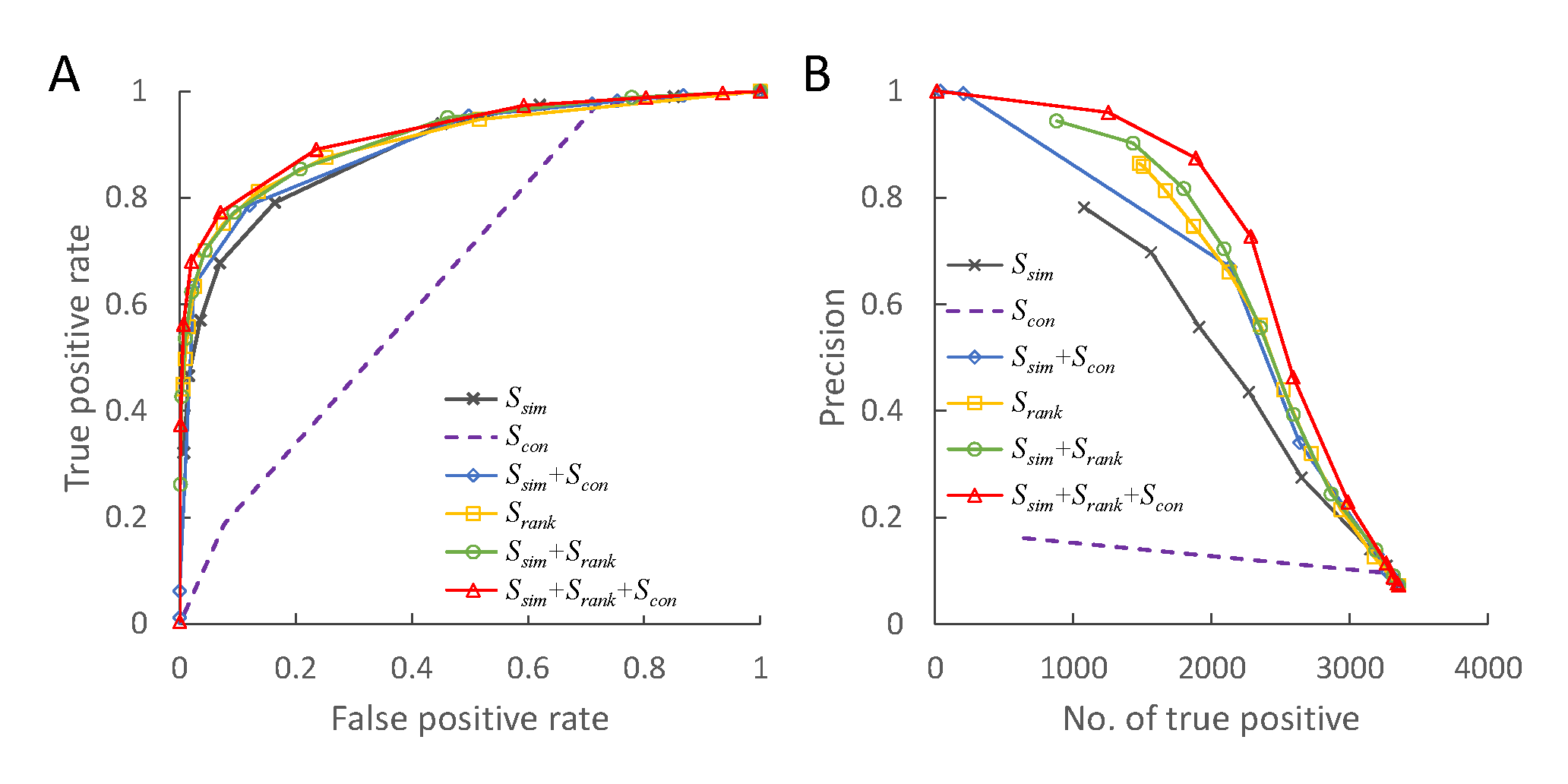

Supplement: S3 Fig — (A) The ROC curves of six scoring combinations, including normalized joint sequence similarity (Ssim), normalized rank (Srank), conserved score (Scon), Ssim + Srank, Ssim + Scon, and Ssim + Srank + Scon, on the MD set for the target organism, M. musculus. (B) The relationship between the number of true-positive cases and the precision of these six scoring combinations. Among these six combinations, Ssim + Srank + Scon is the best. (TIF) [file pone.0116347.s009.tif]

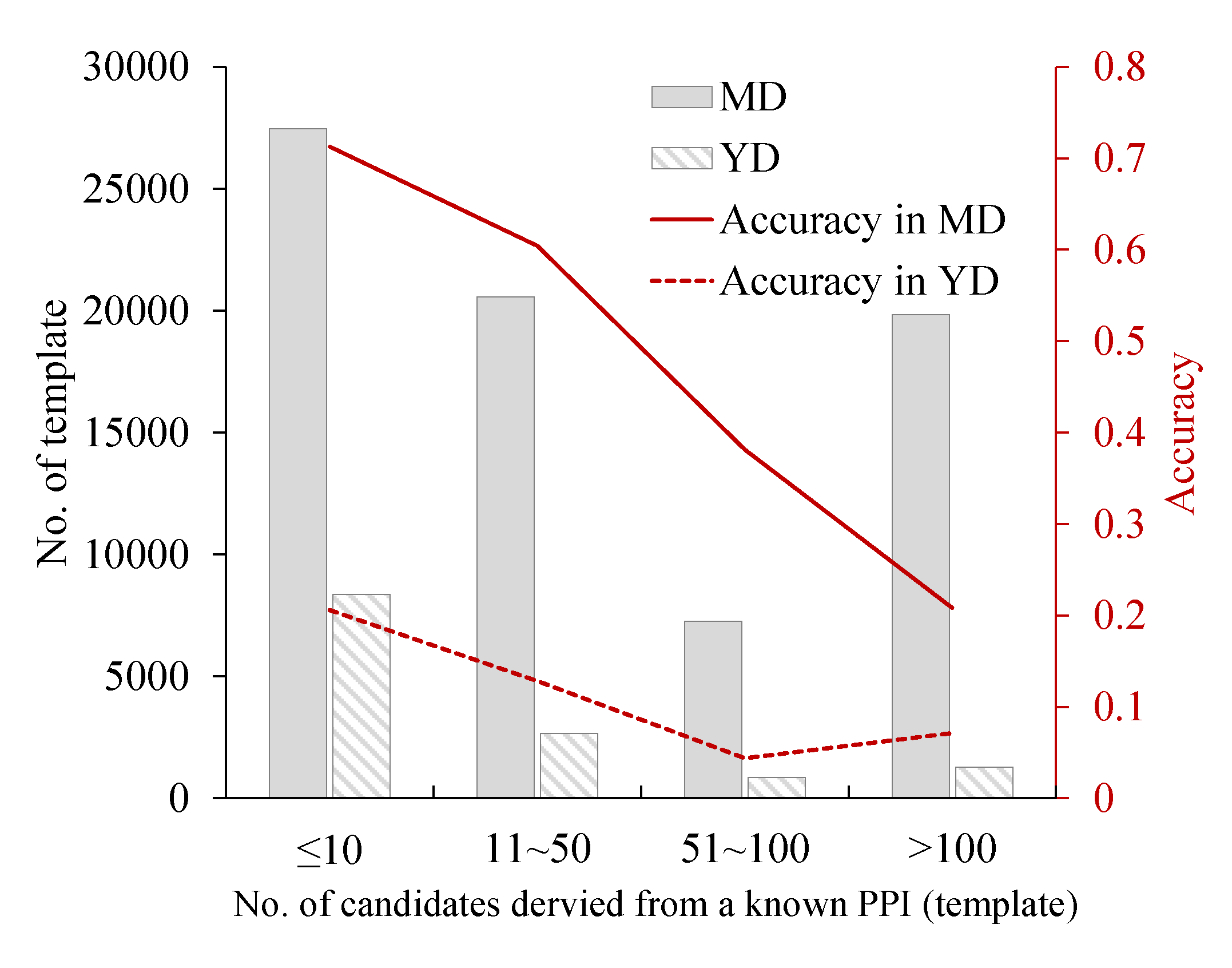

Supplement: S4 Fig — The prediction accuracy of the generalized interologs mapping decreases as the number of PPI candidates increases in both the YD and MD sets. The number of candidates derived from known PPIs increases from S. cerevisiae to M. musculus. (TIF) [file pone.0116347.s010.tif]

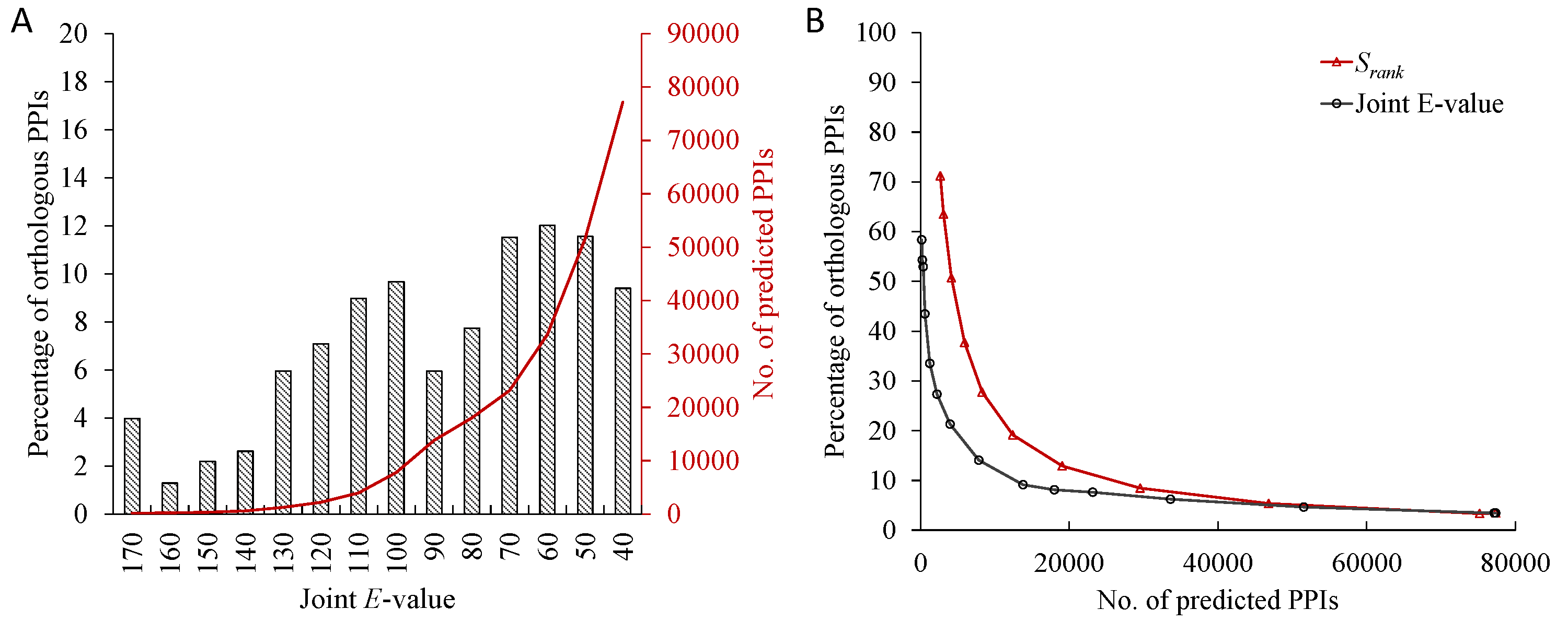

Supplement: S5 Fig — The orthologous interaction means an interacting orthologs protein pair of the template PPI in the source organisms. (A) The distribution of orthologous interactions against the E-value using BLASTP. (B) The distributions of orthologous interactions under different numbers of predicted PPIs derived from the normalized rank (Srank) and sequence similarities (i.e., joint E-value). (TIF) [file pone.0116347.s011.tif]

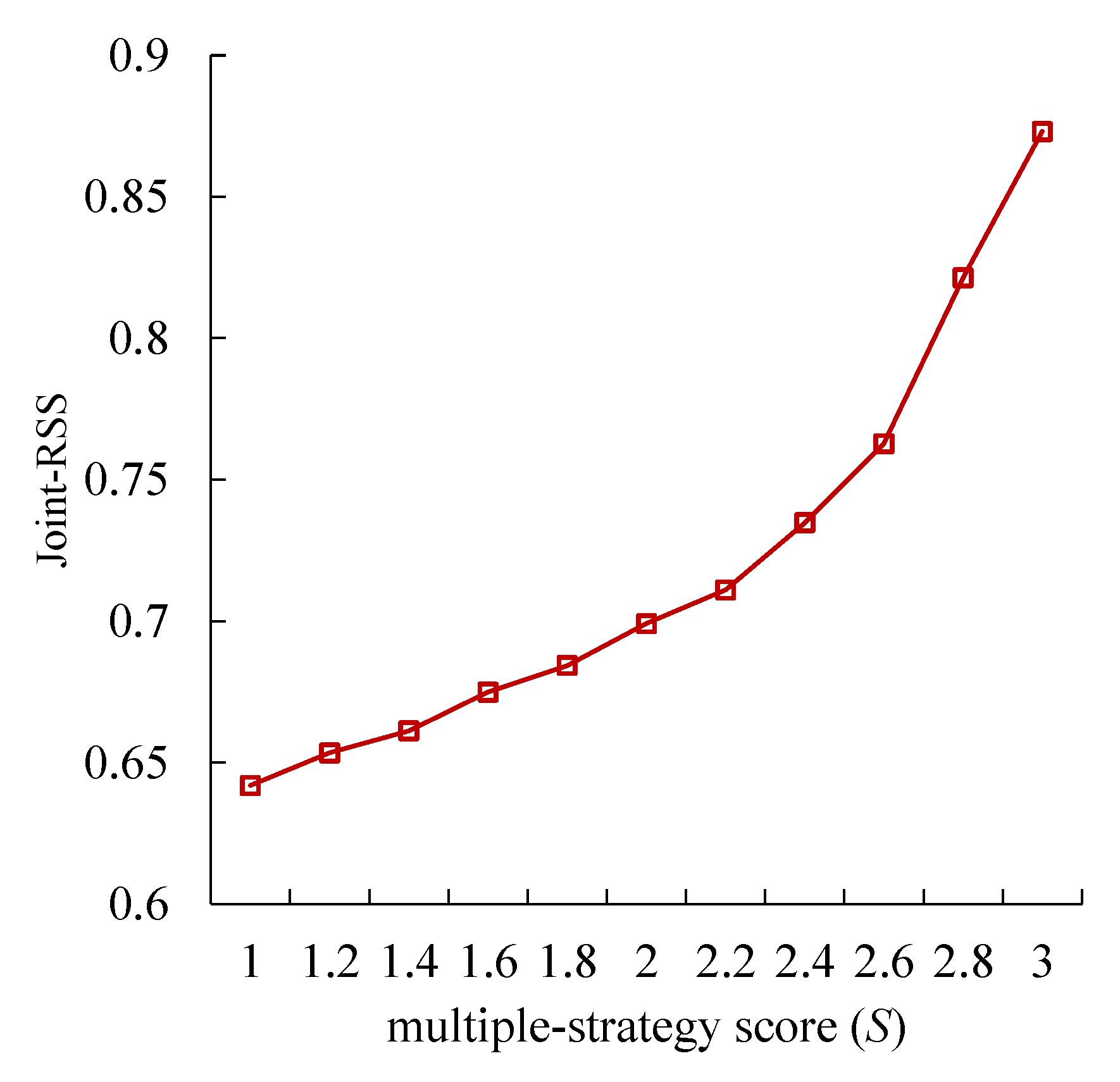

Supplement: S6 Fig — The score (S) is highly correlated with Joint-RSS score. (TIF) [file pone.0116347.s012.tif]

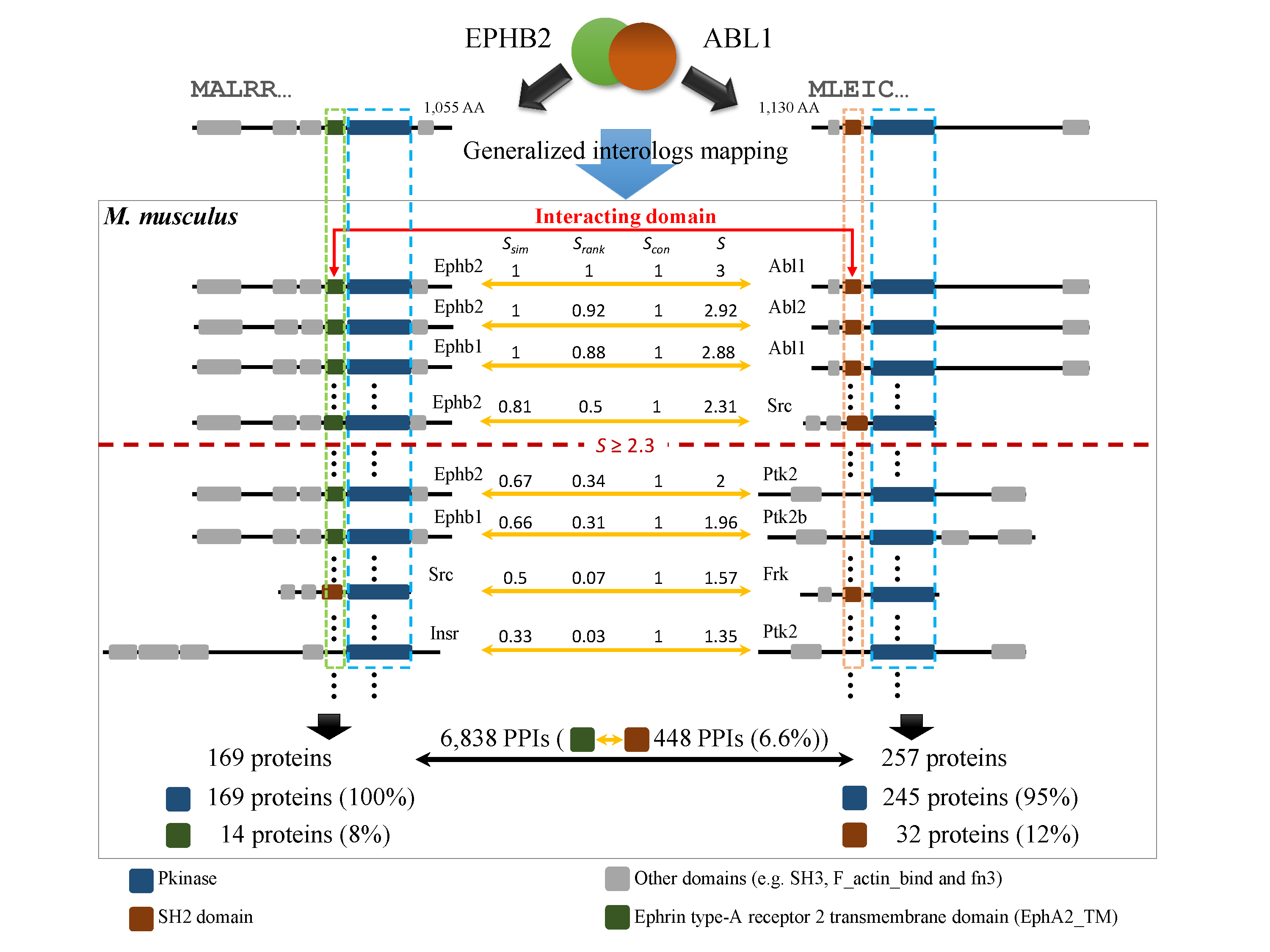

Supplement: S7 Fig — The generalized interologs mapping derived from EPHB2-ABL1 includes 6,838 PPIs. The major domain of both EPHB2 and ABL1 is a kinase domain (blue color). Most homologous proteins of EPHB2 and ABL1 derived from generalized interologs mapping are the kinase domains (100% and 95%, respectively). However, the interacting domains of EPHB2 and ABL1 are the EphA2_TM (green color) and SH2 domain (brown color), respectively. There are only 488 PPIs (6.6%) that retain the interacting domain pairs. (TIF) [file pone.0116347.s013.tif]

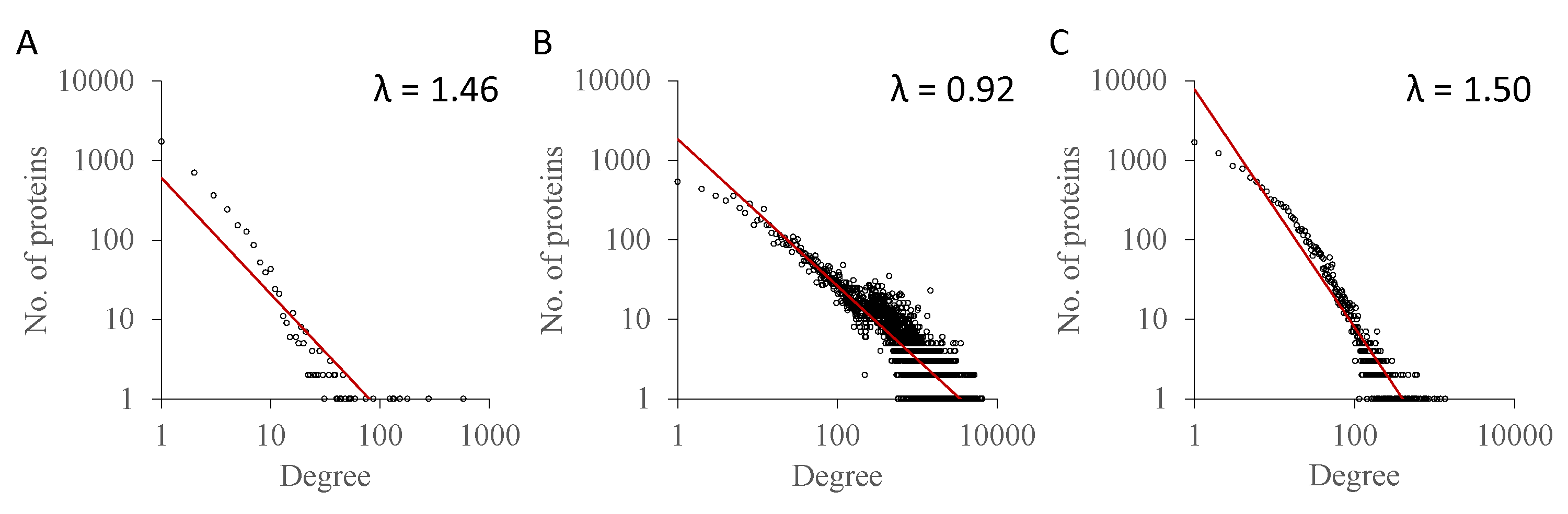

Supplement: S8 Fig — PPI networks derived from experimental PPIs and our method are consistent with the weak scale-free network architectures of some cellular networks. (TIF) [file pone.0116347.s014.tif]

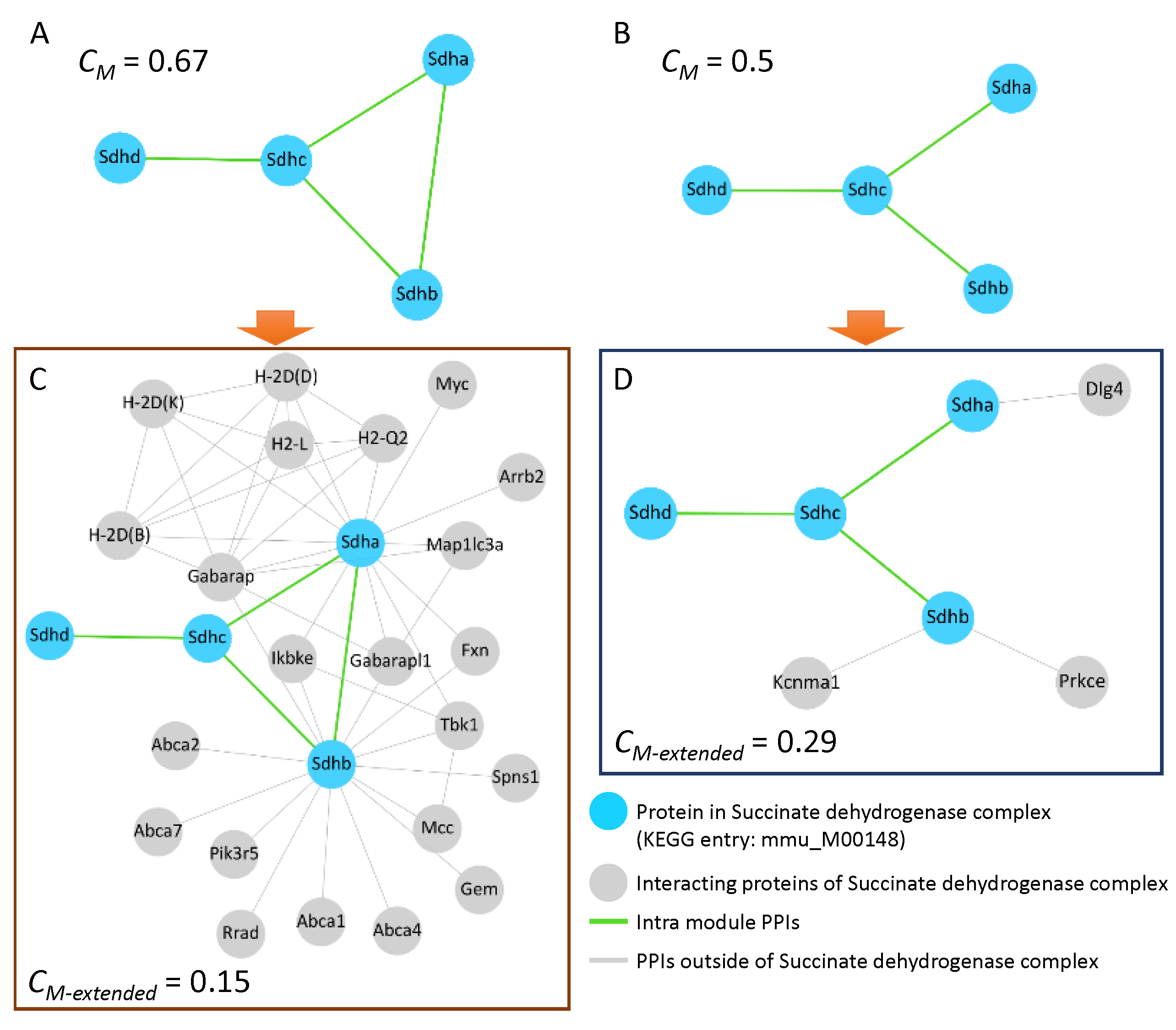

Supplement: S9 Fig — The succinate dehydrogenase complexes have four and three PPIs derived from (A) our methods and (B) experimental PPIs, respectively. The one-layer-extended modules have 49 and 6 PPIs derived from (C) our methods and (D) experimental PPIs, respectively. (TIF) [file pone.0116347.s015.tif]

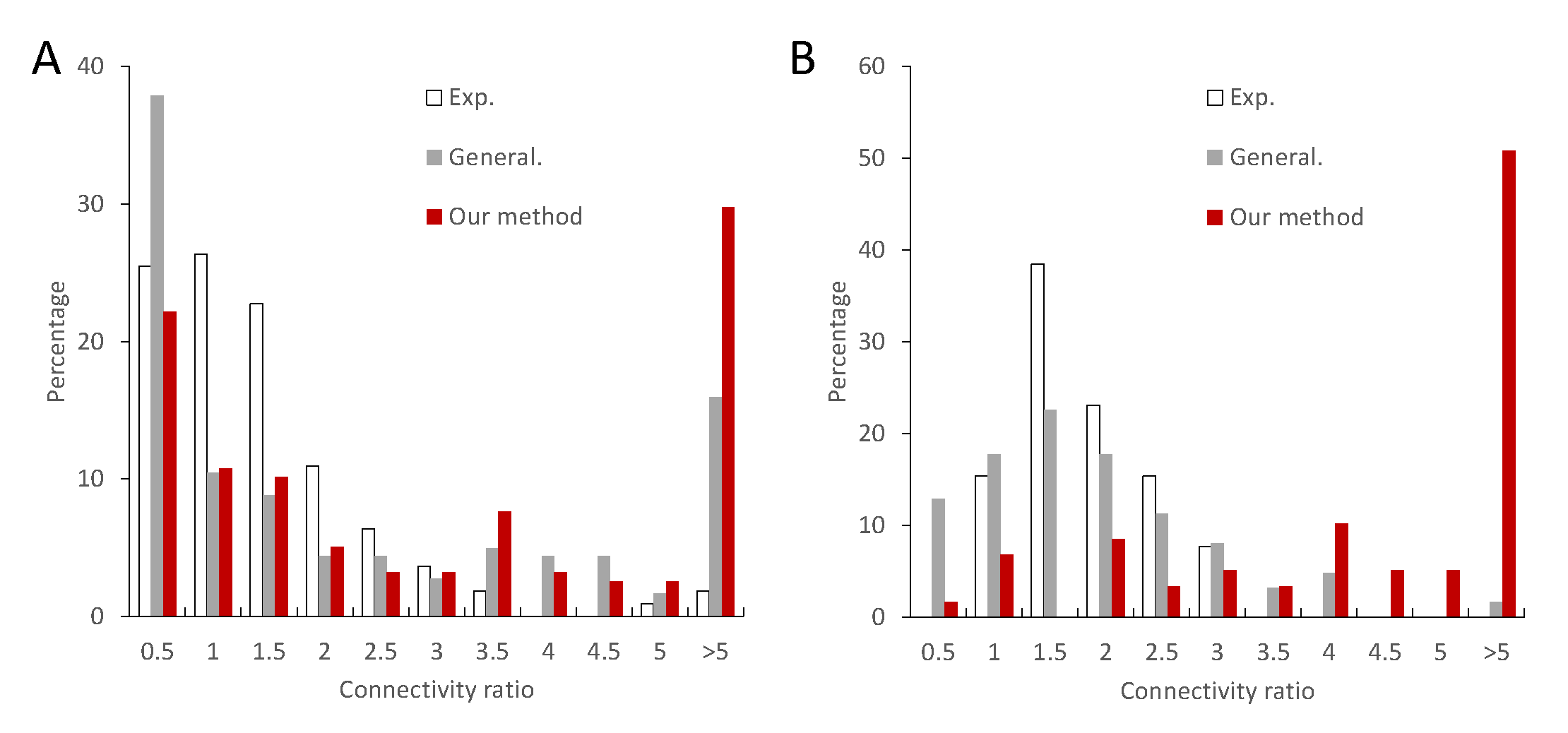

Supplement: S10 Fig — (A) Connectivity ratio of 216 KEGG pathways and one-layer-extended pathways. (B) Connectivity ratio of 76 KEGG complexes and one-layer-extended complexes. The pathways and modules prefer to have highly connected proteins and local compactness (e.g. highly connectivity ratios between modules and one-layer-extended modules) on the networks derived from our methods and experimental PPIs. (TIF) [file pone.0116347.s016.tif]
